# Improved Radio-Cesium Detection Using Quantitative Real-Time Autoradiography

**DOI:** 10.1021/acsomega.3c00728

**Published:** 2023-06-13

**Authors:** Joyce W. L. Ang, Arthur Bongrand, Samuel Duval, Jérôme Donnard, Joni Parkkonen, Satoshi Utsunomiya, Risto Koivula, Marja Siitari-Kauppi, Gareth T. W. Law

**Affiliations:** † Radiochemistry Unit, Department of Chemistry, The University of Helsinki, Helsinki 00014, Finland; ‡ Singapore Nuclear Safety and Research Initiative, National University of Singapore, 138602 Singapore; § AI4R, 2 rue Alfred Kastler, 44307 Nantes, France; ∥ IMT Atlantique, Nantes Université, CNRS, SUBATECH, F-44000 Nantes, France; ⊥ Department of Physics, University of Jyväskylä, Jyväskylä 40500, Finland; # Department of Chemistry, Kyushu University, 744 Motooka, Nishi-ku, Fukuoka 819-0395, Japan

## Abstract

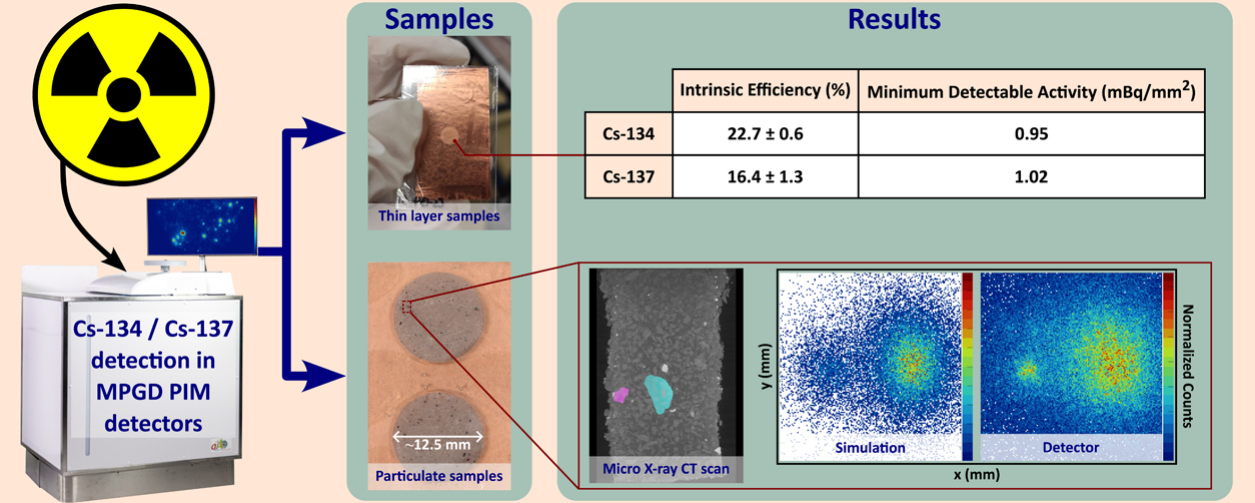

Cesium-134 and -137 are prevalent, long-lived, radio-toxic contaminants released into the environment during nuclear accidents. Large quantities of insoluble, respirable Cs-bearing microparticles (CsMPs) were released into the environment during the Fukushima Daiichi nuclear accident. Monitoring for CsMPs in environmental samples is essential to understand the impact of nuclear accidents. The current detection method used to screen for CsMPs (phosphor screen autoradiography) is slow and inefficient. We propose an improved method: real-time autoradiography that uses parallel ionization multiplier gaseous detectors. This technique permits spatially resolved measurement of radioactivity while providing spectrometric data from spatially heterogeneous samples—a potential step-change technique for use after nuclear accidents for forensic analysis. With our detector configuration, the minimum detectable activities are sufficiently low for detecting CsMPs. Further, for environmental samples, sample thickness does not detrimentally affect detector signal quality. The detector can measure and resolve individual radioactive particles ≥465 μm apart. Real-time autoradiography is a promising tool for radioactive particle detection.

## 1 Introduction

Cesium-134 and -137 are fission product radionuclides that are often released into the environment from the civil nuclear industry (e.g., from nuclear power plants during routine operations, reactor accidents, etc.). Cs isotopes are also released during nuclear weapon tests.^1−3^ Due to cesium’s volatility and the relatively long half-lives of Cs-134 and Cs-137 (2.0652 and 30.08 years, respectively), radio-Cs isotopes present an environmental concern as they are often a significant contributor to radiation doses in areas impacted by nuclear fallout.^3,4^

Radio-Cs can be discharged into the environment in the form of aerosol, soluble aqueous solution, or as part of sparingly soluble particles.^5−8^ Significant amounts of Cs were released into the environment during the 1986 Chernobyl (132 PBq) and the 2011 Fukushima Daiichi nuclear power plant accidents (32 PBq).^9−11^ Atmospheric release of radio-Cs aerosols results in plumes of radio-Cs, which are distributed by air-mass movements. Radio-Cs in the plumes is subsequently brought to the ground by wet deposition via precipitation or dry deposition.^12,13^ Since radio-Cs has a high affinity for clay minerals, it often undergoes sorption to clayey soil particles.^14,15^ In addition, plants can uptake Cs from air or water,^16,17^ introducing radio-Cs into the biosphere, which dominates dose in the impacted regions.

After the Fukushima Daiichi nuclear power plant (FDNPP) accident, which provides motivation for this study, radio-Cs derived from atmospheric fallout was presumed to have become largely associated with clay minerals in regional soils.^14^ As a result, early remediation efforts focused on removing the uppermost centimeters of the soil profile in contaminated areas. The contaminated soils were then transported for interim storage in local facilities.^18^ However, in 2013, discrete microparticles containing very high specific activities of radio-Cs were found in air filters and regional soils.^19^ The Cs-rich microparticles are now known in the literature as CsMPs.^19,22,23^ These particles were generally in the micron size range with specific activities of ∼10^10^ to 10^11^ Bq/g (10^7^ times higher than radio-Cs sorbed onto clay minerals in the FDNPP exclusion zone),^20^ and subsequent work has shown them to be sparingly soluble.^4,21^ Due to their size, CsMPs have the potential to be inhaled and possibly retained in the human lung.^21,24^ Inhalation of CsMPs could expose humans to an internal dose. Recent studies on CsMP exposure to human primary lung fibroblast and bronchial epithelial cell lines have shown that the radiation dose from a CsMP induces inflammatory signaling and DNA damage responses within 24 h.^25^ In addition, some radioactive microparticles from the FDNPP contain very high concentrations of radio-Cs that could potentially provide discrete external dose to the skin.^23^ Based on the dose calculations, 0.5–1 h skin contact with such particles can potentially cause skin lesions.

Research by Ikehara et al. has shown that a substantial amount of CsMPs were released from the FDNPP and contaminated a widespread area. They found that the fraction of total Cs radioactivity in environmental samples associated with CsMPs, collected from 20 sites, ranged from 1.63 to 80.2%. Further, in the same samples, the number of CsMPs in the soils ranged from ∼1 to 318 particles per gram of soil.^26^ Moreover, CsMPs can be transported (e.g., as dust in the air, by flowing water, etc.), resulting in an unpredictable, changing radio-Cs distribution in the environment.^27−30^ Given the existence of CsMPs and their potential long-term impact on the environment and human health, it is essential to detect, evaluate, and differentiate the varying forms of radio-Cs (micro-particle or bulk) found in samples.

Current methods used for the detection of radio-Cs in environmental samples include γ spectroscopy and autoradiography. γ spectroscopy is routinely used for the measurement of bulk radio-Cs activity concentrations.^16,31^ Use of the peak energies at 604.7 keV (Cs-134) and 661.7 keV (Cs-137) also enables accurate measurement of Cs-134/Cs-137 isotopic ratios, which can be used to identify the origin of Cs releases.^32^ Autoradiography is an imaging technique that provides high-resolution two-dimensional images of radioactive emissions from a sample. Analysis of air filter samples taken during the FDNPP accident with phosphor screen autoradiography first highlighted the presence of CsMPs.^22^ Autoradiography has also been applied to soil samples to show the presence of CsMPs,^4,19^ and it has been used to differentiate between Cs emissions from clay minerals in soils and CsMPs via the “quantification of CsMPs” (QCP) method.^20^ Subsequently, the QCP method has been used to quantify and map the amount of CsMPs located in different soil samples collected in Japan.^26^ Contaminated filter or soil samples were exposed to an imaging plate to obtain the spatial positions of the radioactive particles.^19,22^ Thereafter, the sample near the hotspot is extracted and prepared for another round of autoradiography. The process of autoradiography and extraction is repeated until there are insignificant amounts of sample other than CsMPs left.

The current technique of sample-splitting using phosphor screen autoradiography is laborious and time-consuming. Moreover, it is essential to get the exposure time of the samples to the imaging plate correct to prevent over- or underexposure. This is difficult and prone to error for samples of unknown activities, resulting in repeated, time-consuming measurements with different exposure times. Another limitation of phosphor screen autoradiography is the inability to perform nuclear spectrometry directly. This technology does not permit energy-based count separation, thus making it impossible to separate or identify different radionuclides in the same sample.

Real-time autoradiography in direct counting mode using micro-pattern gas detectors (MPGDs) has been developed.^33^ It can eliminate the problem of sample over- or underexposure as faced in phosphor screen autoradiography. An example of such a detector is the BeaQuant. It employs use of a parallel ionization multiplier (PIM) structure (thin sandwich of two metallic micromeshes) to achieve pre-amplification close to the sample surface.^34^ For such techniques, the autoradiograph can be immediately seen during the measurement and the sample’s radioactivity can be gauged without stopping the acquisition. In contrast to phosphor screen autoradiography, which provides an image that is indirectly measured by the photo-stimulated luminescence of the imaging plate, real-time autoradiographs construct the image by directly measuring the individual decay emitted from the sample.^35^ Due to the low density and thickness of the active gas media, MPGDs are largely insensitive to γ radiation at room temperature and atmospheric pressure. Furthermore, MPGDs could be used to identify individual radionuclides in the sample/autoradiograph as they record the energy of the emitted radiation.^36^ Here, the energy difference between different emissions needs to be substantial. Given the advantages of real-time autoradiography with an MPGD over phosphor screen autoradiography, we explored the limitation of the detector, improved our understanding in applying MPGDs to samples containing radio-Cs, and discussed its possible application for monitoring of environmental radioactivity.

**Figure 1:**
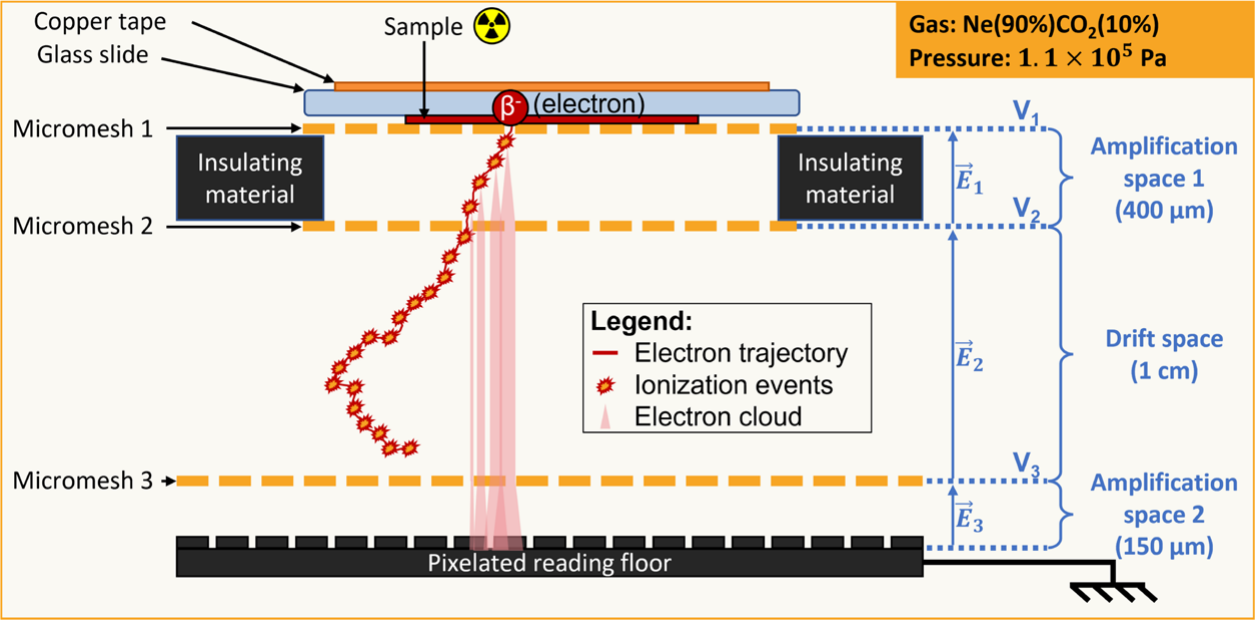
Schematic diagram showing a cross section through the BeaQuant (using a GS12-08 sample holder of size 120 mm × 80 mm). The components of the detector are contained in a gas mixture of 90% neon and 10% carbon dioxide, at a pressure of 1.1 × 10^5^ Pa. The electron trajectory in red shows an example of an emitted β^−^ particle and its interaction with the gas within the chamber of the detector.

Figure 1 shows a schematic diagram of a BeaQuant detector showing its working principles. A sample is loaded onto a sample holder (labeled as “micromesh 1” in Figure 1). The BeaQuant (Figure 1) is made up of two to three (depending on the sample holder) stainless steel woven micromeshes, which have a high voltage applied to them. As a result, an electric field is produced between the meshes, producing two different types of space: an amplification space (with a higher electric field) and a drift space (with a lower electric field). When a Cs-134 or -137 atom emits a β^−^ particle, the particle will travel through the detector and undergo ionization through interaction with the detector gas, thus forming an ion–electron pair. During an ionization event in the amplification space, electrons will undergo an avalanche, which amplifies into an electron cloud. In the drift space, the electrons will not be amplified, instead, they will travel toward the reading floor. Ultimately, the reading floor records the electron-cloud-induced signals, which are then used for image reconstruction.

Although the employment of MPGDs in the detection of radio-Cs is attractive, theoretical understanding of the detector properties and response to radio-Cs must be improved to make a sensible, reliable analysis of environmental samples. Detection limits are required to reliably (with a level of significance) differentiate between the presence or absence of radio-Cs in a sample. Further, in order to determine if a PIM gaseous detector can separate radio-Cs isotopes (i.e., Cs-134 from Cs-137) by deconvolution, the energy deposition of radio-Cs isotopes in the detector must be evaluated. Finally, sample thickness will play an important role in affecting the signal quality of autoradiography. In a real-life environmental sample (for example, CsMPs in soil), radio-Cs atoms that are not at the sample surface will produce a greater spread of data and lower intensity.

Reflecting the above, the objective of this study was to generate a framework for measuring radio-Cs in environmental samples with the PIM structure, using Monte Carlo simulations with a GEANT4 toolkit and Cs-134 and Cs-137 samples of known radioactivity. To achieve this, we sought to evaluate the detection limits of a detector configuration, explore the possibility of separating Cs-134 and Cs-137 radioisotopes based on their energy spectrum by deconvolution, and examine the effects of sample thickness on the signal of the autoradiograph acquired.

## 2 Results and Discussion

### 2.1 Novel Radio-Cs Standards for Detector Assessment

Two novel types of radio-Cs standard samples, thin-layer (to minimize self-absorption of emitted radiation) samples, and Cs-134 labeled particles, respectively, were synthesized. The thin-layer samples were used as a simplified standard to obtain the detection limits of the detector as well as to test the deconvolution of Cs-134 and Cs-137 emissions from the same sample. The Cs-134 labeled particles were used to simulate real-life environmental radio-Cs particles (e.g., CsMPs) in samples of varying thicknesses. More details on sample synthesis can be found in Section 4.

### 2.2 Detection Efficiency and Limits

The intrinsic efficiency of the BeaQuant system for both Cs-134 and Cs-137 was determined using the calibration curves acquired from thin-layer samples (Figure S1) and the computed fraction *F*_e_ from our simulation, where
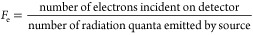
1

The intrinsic efficiency ϵ_int_, defined as the ratio of the number of pulses recorded to the number of radiation quanta incident on the detector, is given by
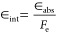
2where ϵ_abs_ is the absolute efficiency and *F*_e_ is the fraction of electrons incident on the detector over the total β particles emitted from sample. The values of fraction *F*_e_ and the efficiencies (ϵ_abs_ and ϵ_int_) for both Cs-134 and Cs-137 are listed in Table 1.

**Table 1:** Fraction *F*_e_ and Efficiencies (ϵ_abs_ and ϵ_int_) for the Detection of Cs-134 and Cs-137 with the BeaQuant System^a^

radioisotope	ϵ_abs_ (%)	*F*_e_	ϵ_int_ (%)
Cs-134	11.94 ± 0.32	0.526	22.7 ± 0.6
Cs-137	11.08 ± 0.83	0.675	16.4 ± 1.3

a The fraction *F*_e_ (rounded off to 3 sig. fig.) was calculated using eq 1, the absolute efficiency was calculated by averaging the ratios of all seven data points from the calibration curves (Figure S1), and the intrinsic efficiency was calculated using eq 2. Uncertainty for absolute efficiency represents the standard error for the seven samples, while uncertainty for intrinsic efficiency was calculated using error propagation.

The intrinsic efficiency of the detector is independent of sample geometry or exposure time; it primarily depends on the detector’s properties and radiation energy. Hence, given the intrinsic efficiencies reported in Table 1, coupled with computations of fraction *F*_e_ (which accounts for sample geometry), we can calculate the activities of any Cs-134 or Cs-137 samples so long as the sample geometry is known. Moreover, since the detector has a linear response, quantification of the Cs activities can be done without measuring the calibration standards simultaneously with the samples. This is an advantage over the traditional phosphor screen autoradiography, where the calibration standards must be exposed alongside the samples for quantitative measurement. In addition, the absolute efficiency of the BeaQuant system (11.94 and 11.08% for Cs-134 and Cs-137, respectively) is higher than γ spectroscopy (4.98 and 4.59% for Cs-134 604.7 keV peak and Cs-137 661.7 keV peak, respectively) for the same sample.

Polymerized-methyl methacrylate (PMMA) standards have already been used for the calibration of the PIM gaseous detector.^35,37,38^ The thickness of PMMA standards is important to account for the sample density and radioactive particle range in porosity studies of bulk samples. However, the application of PMMA standards into radioactive surface studies requires an extra computational step to convert bulk volume activity concentration to surface activity concentration.^37^ On the other hand, thin-layer samples are more suitable for radioactive surface studies, such as surface contamination or Cs-containing particles in thin sections. The thickness of the PMMA standards results in a more complicated geometry compared to thin-layer samples. In thin-layer samples, there is minimal loss in electron energies due to their thin nature. In addition, the sample preparation for the thin-layer standards is less time-consuming and safer compared to PMMA standards, which require sawing and polishing of radioactive materials after the polymerization process. The high coefficient of determination for the linear fit (Figure S1) obtained from the Cs-134 and Cs-137 calibration curves (*R*^2^ = 0.996 and 0.998, respectively) proves that the thin-layer standards are a viable option for calibration of radioactive surface studies.

Currently, there are no efficiency calculations for the detection of radio-Cs using this type of MPGD found in other studies. As a result, the efficiency attained in this paper cannot be validated. However, in the future, this method of sample preparation and analysis can be applied to other radionuclides of interest to obtain their intrinsic efficiencies. These values could then be cross-referenced to other studies (e.g., efficiency for carbon-14 = 36% and efficiency for barium-133 = 82.1%) to evaluate the validity of this method.^35,38^

Our study also evaluated the critical level *L*_C_, detection limit *N*_D_, and minimum detectable activity MDA per unit area for the detection of radio-Cs using the BeaQuant system. The blank sample was used to acquire the background counts from the detector. The background counts were substituted into the equations, which were adapted from Currie’s formulation.^39^ The formulae used to calculate these variables can be found in the Supporting Information (Text S1). In the BeaQuant with an acquisition of time *t* = 17 h, we obtained background counts per unit area of *N*_B_ = 9.66 ± 0.10 counts/mm^2^ for an area of *A* = 1056 mm^2^. *L*_C_ was then calculated to be 0.223 and *N*_D_ 6.93 counts/mm^2^. As a result, the MDA for Cs-134 and Cs-137 were 0.947 and 1.02 mBq/mm^2^, respectively. The values for *L*_C_ and *N*_D_ meant that for an acquisition time of 17 h, any net counts below 0.223 counts/mm^2^ will be considered background (no presence of radioactivity) with a false-positive probability of 5%, whereas any net counts above 6.93 counts/mm^2^ will imply that there is radioactivity with a false-negative probability of 5%. From the MDA values, we established that only Cs-134 samples containing a surface activity concentration >0.947 mBq/mm^2^ can be detected by the BeaQuant system with a 95% confidence. Similarly, only Cs-137 samples with a surface activity concentration of >1.02 mBq/mm^2^ can be detected with a 95% confidence.

To assess the feasibility of MPGDs in the detection of radioactive particles, the *N*_D_ and MDA were also calculated and expressed per unit μm^2^ using the same background acquisition data. The *N*_D_ was calculated to be 2.71 counts/μm^2^, while the MDA was 0.370 mBq/μm^2^ and 0.399 mBq/μm^2^ for Cs-134 and Cs-137, respectively. CsMPs emitted from the FDNPP accident were reported to have activities of >60 mBq per particle.^20^ Here, we take the extremity by calculating the surface activity concentration for a 5 μm radius CsMP with 60 mBq. The projected surface activity concentration for that particle is 0.764 mBq/μm^2^, which is higher than the MDA. This example illustrates the capability and potential of MPGDs for detecting CsMPs or other Cs-containing microparticles.

### 2.3 Deconvolution of Cs-134 and Cs-137

To assess the possibility of separating radioactive emissions from Cs-134 and Cs-137 atoms, we compared the electron energy spectra of Cs-134 emission with that of Cs-137. The BeaQuant system detects electrons, including Auger and conversion electrons. Table S1 summarizes the type of electrons and their energies emitted from Cs-134 and Cs-137.^40^

**Figure 2:**
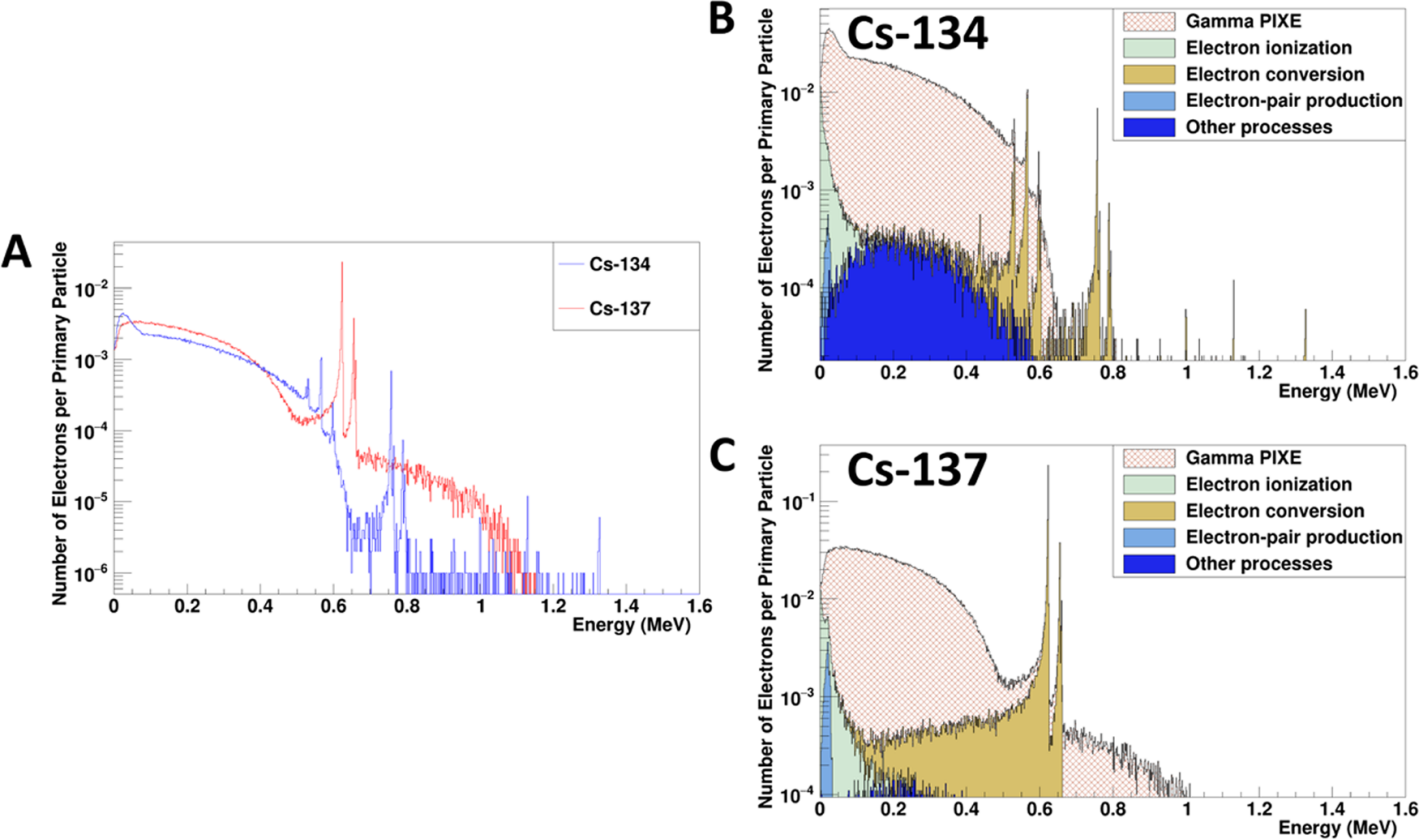
Computed electron energy spectra from Cs-134 and Cs-137. (A) Comparison of the electron energy found on the sample surface (in contact with the detector) for Cs-134 and Cs-137 emissions. The breakdown in terms of different electron energy contribution from various physics processes (based on GEANT4) is shown for (B) Cs-134 and (C) Cs-137, respectively. Other processes include the photoelectric effect, Auger electrons, and Compton scattering. The values in the *y*-axis (number of electrons per primary particle) were obtained via the division of the number of events recorded in each energy bin by the total number of decay events simulated (1,000,000).

To further aid the understanding of the electron energy distribution, GEANT4 (with the geometry of the thin-layer samples) was used to plot and compare the electron energies for Cs-134 and Cs-137 emitted onto the sample surface (Figure 2).

The electron energy distributions from the γ particle-induced X-ray emission (PIXE) contribution shown in Figure 2B,C agreed with the β decay spectra for both Cs-134 and Cs-137.^41,42^ This demonstrates that the GEANT4 model for Cs-134 and Cs-137 are accurate. From Figure 2A, we can observe that there are two main differences in electron energies between Cs-134 and Cs-137. First, the conversion electrons in Cs-137 are more distinct. Second, there are significantly higher counts for Cs-137 electrons in the energy region of 0.8 to 1 MeV compared to Cs-134.

Although there is a theoretical difference between the two spectra, the ability to separate the Cs radioisotopes ultimately depends on the differences in electron energy recorded by the detector. Therefore, we needed to investigate the electron energy deposition in the MPGD’s amplification space 1.

The energy deposition depends heavily on the incident electron energies, as well as the influence of the amplification gain. As explained in the working principles of the BeaQuant system, electrons passing through the amplification space will undergo ionization, thereby producing a series of ionization events in one electron trajectory (see Figure 1). The ionization event would cause an avalanche, which amplifies into an electron cloud. Therefore, in each ionization event, the electron avalanche deposits a cumulative energy *E* into the detector
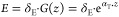
3where δ_*E*_ is the energy deposited by the single passing electron in the ionization event and *G*(*z*) is the gain experienced by the electron. The gain can be expressed as a function of *z*: the vertical distance between the ionization event and micromesh 2. To determine the actual gain, the Townsend coefficient α_T_ (which depends on several factors such as the electric field in the amplification space, the gas pressure, and its composition of gas in the detector) must be evaluated.

Given that a single electron trajectory produces multiple ionization events, the total energy deposited by an incident electron into the detector is given by
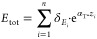
4where δ_*E*_*i*__ and *z*_*i*_ are the energy deposited and vertical distance recorded for the *i*^th^ ionization event, respectively, and *n* is the total number of ionization events in the single electron trajectory.

The GEANT4 simulation seeks to incorporate the amplification gain as shown in eq 4. To achieve this, the Townsend coefficient was estimated. For a gas detector with a gas composition of Ne(90%)CO_2_(10%), a gas pressure of 1.1 × 10^5^ Pa, and an amplification space electric field of 1.95 × 10^4^ V/cm, the Townsend coefficient was estimated to be 35 mm^–1^ from computations by Donnard (2008).^43^ Using this value, the total energy deposited by Cs-134 electrons and Cs-137 electrons into the detector was simulated.

**Figure 3:**
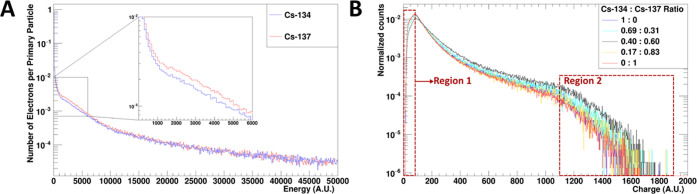
Electron energy deposited in the detector. (A) GEANT4 simulation of electron energy deposited in amplification space 1 from a Cs-134 and Cs-137 thin-layer source. The number of electrons per primary particle was obtained by dividing the number of events recorded in each energy bin by the total number of decay events simulated (1,000,000). (B) The charge distribution acquired from the BeaQuant system for thin-layer samples with 5 varying ratios of Cs-134 to Cs-137. To normalize the counts in (B), the counts in each charge bin were divided by the total number of counts in the sample recorded by the detector. Since the BeaQuant is working as a proportional gas counter, the charge measured on the readout plane in the *x*-axis of (B) is directly proportional to the energy deposited into the amplification space in the *x*-axis of (A). As a result, they can be compared directly. The dashed rectangular boxes in red highlight the regions where there are deviations between the simulation results and experimental data.

In addition to the simulations, experimental data were collected from the thin-layer samples with 5 varying ratios of Cs-134 to Cs-137 using the BeaQuant system. The total energy deposited per electron is represented by the charge recorded by the detector per event. The simulation and experimental data are compared in Figure 3.

Based on the simulation and experimental data, we noted that there was no significant difference between the energy deposited by sources of Cs-134 and Cs-137 in the detector. In fact, the only difference was from the slight divergence at energies between 1000 to 6000 au as shown in Figure 3A. This could be attributed to two reasons. First, the contribution from the amplification gain may have caused the distinct conversion electron peaks from Cs-137 to become less defined. As a result, the conversion electron peak only contributed to the divergence. Second, there are too many overlaps in the electron energy distributions for the Cs-134 and Cs-137 sources (as shown in Table S1 and Figure 2). Higher-energy electrons tend to have lower deposition (ionization events) in the amplification space compared to electrons with lower energy. Hence, the presence of Cs-137 electrons from 0.8 to 1 MeV (Figure 2) should be reflected in the lower deposition energies. There should be higher counts in lower deposition energies (e.g., *E*_tot_ < 6000 au) for Cs-137 compared to Cs-134, which accounts for the divergence (1000–6000 au) as well. However, this lower deposition energy region is also dominated by low-energy electrons that stop within the amplification space. Therefore, although there were differences in the electron energy spectra for Cs-134 and Cs-137, the contributions from the inherent electron energy difference were insignificant.

Comparison between Figure 3A,B shows that the GEANT4 simulations and experimental data are mostly in agreement since the plots follow a similar general trend. This demonstrates that the GEANT4 simulation is a reliable approach to understand the basic detector function response. There are some differences in the plots, as demarcated by region 1 and region 2. We postulate that these differences are due to the detection threshold and the dynamics of the detector electronics for higher- and lower-energy depositions. Moreover, the theoretical divergence cannot be identified in Figure 3B, most likely due to the fluctuations from both counting statistics and electronics. Thus, with the current technology and electronics, we surmise that the slight divergence in energy deposition cannot be used to distinguish the Cs-134 from Cs-137 radioisotopes accurately. While it is not currently possible to separate Cs-134 from Cs-137, it might be worthwhile for future studies to consider the possibility of separating the emissions of radio-Cs from other interfering β emitting radionuclides, for example, technetium-99, which has also been found in CsMPs.^44^

### 2.4 Effect of Sample Thickness

**Figure 4:**
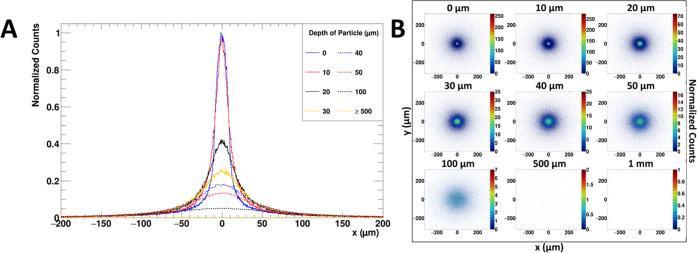
Simulated spatial distribution for radioactive Cs-134 particle from different depths. (A) Surface spatial distribution (in the *x*-axis) for electron emitted from a Cs-134 particle (10 μm radius, spherical) placed at varying depths from the sample surface. The counts were normalized by the maximum peak height at depth = 0 μm. (B) Scatterplot for each respective depth.

To understand the effect of sample thickness on data fidelity/quality, it is necessary to study the trajectories of the β particles emitted from the radioactive samples as they make their way toward the detector. However, since the emitted β particles (electrons or positrons) are charged particles and have a lower mass relative to atoms (∼0.000543 atomic mass unit), they will be strongly scattered within the traversed medium. As a consequence, their trajectories are unpredictable and typically nontrivial. In this study, we utilized both GEANT4 and BeaQuant acquisition of resin-embedded particulate samples with three different thicknesses (10s of μm, 100s of μm, and 1 mm). GEANT4 was used to simulate the spatial distributions of electrons incident on the detector emitted from a Cs-134 containing micro-particle, which was placed at varying depths (0, 10, 20, 30, 40, 50, 100, 500 μm, and 1 mm) from the sample surface. Figure 4 shows the 1-dimensional spatial plot of the electron and the scatterplot for each particle depth.

Within the first 10 μm of depth (Figure 4A), there are insignificant differences in the signal, albeit there are some slight changes in the spread of the distribution. In contrast, once the particle is 20 μm away from the sample surface, the peak height decreases drastically to less than half of the original height, and the peak is broadened. To quantify the broadening of the peaks, the full width at half-maximum (FWHM) of each peak was computed (Table 2).

**Table 2:** FWHM Obtained from Each Simulated Peak Shown in Figure 4

depth (μm)	0	10	20	30	40	50	100	≥500
FWHM (μm)	17.2	18.4	37.2	58.8	76.4	94.8	182.8	375.2

The FWHM of a peak can be used to evaluate the spatial resolution. Taking the example of a 10 μm radius Cs-134 particle on the surface (depth = 0 μm), the FWHM is 17.2 μm, which implies that in a cluster of particles, each particle must be at least 17.2 μm apart to be seen as a distinct particle. From the values in Table 2, we note that when the particle is 20 μm away from the sample surface, the FWHM is more than double that of the particle found on the sample surface. These findings suggest that the signal degrades within the first 20 μm of the sample surface.

Further observations from Figure 4B indicate that particles beyond 50 μm of depth contribute to a blurred signal that can be interpreted as noise. Hence, we can infer that the most crucial depth for the sample is within the first 50 μm. Regardless of the sample thickness, the majority of peaks recorded by the detector will originate from particles within the first 50 μm of the sample; any other particles will contribute to the background counts instead. By extension, it would mean that the sample thickness does not affect the counting of the peaks, but only the background signal. Moreover, to only count the particles within the 50 μm thickness, unwanted peaks can be filtered out by setting an upper FWHM threshold of 94.8 μm, which corresponds to the 50 μm mark.

**Figure 5:**
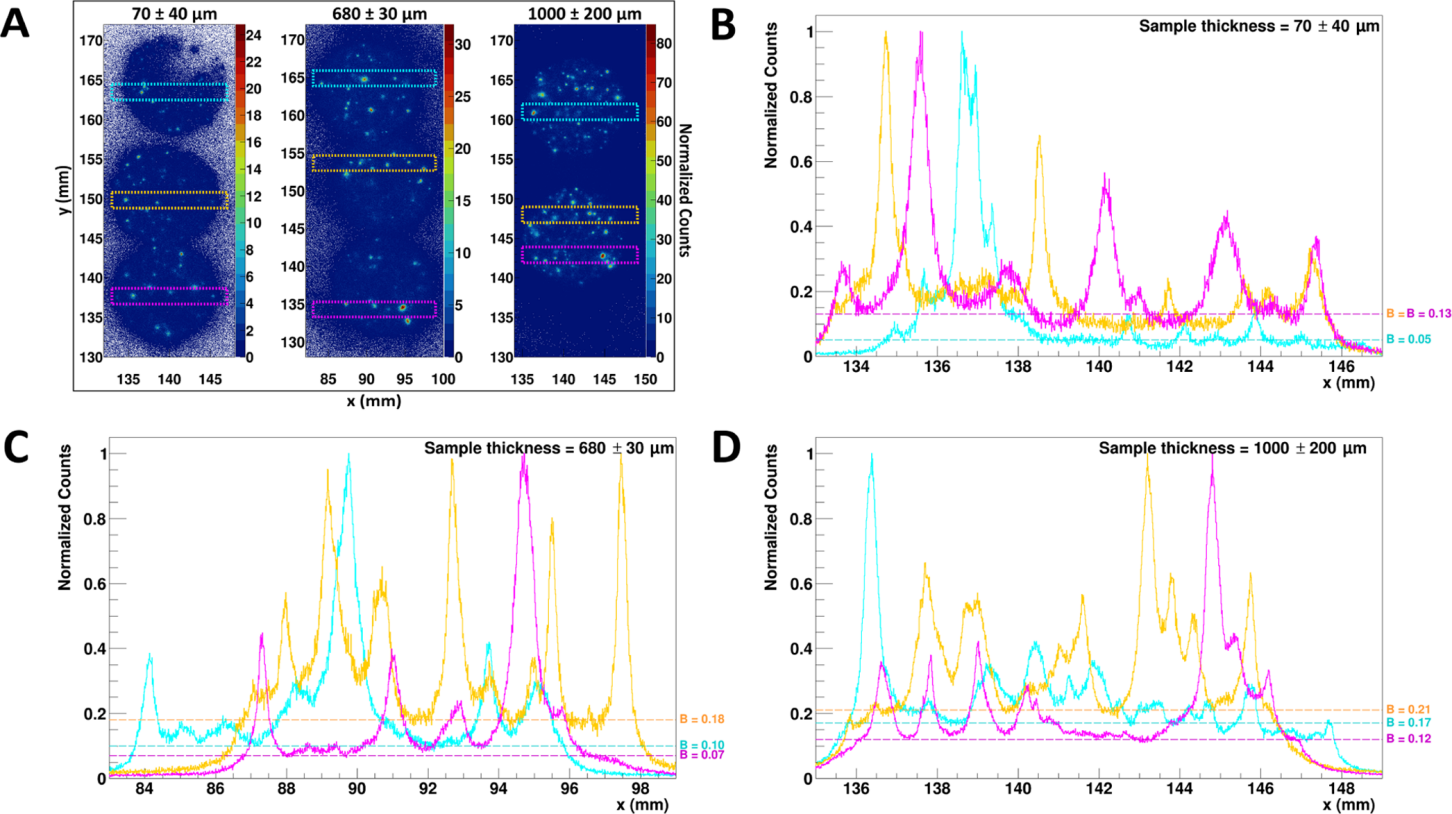
BeaQuant data for samples with different thicknesses. (A) Autoradiograph (10 μm × 10 μm per pixel) of three different samples and the spatial distribution for the regions of interest (cross-section width of 2 mm) found in the (B) 70 μm, (C) 680 μm, and (D) 1000 μm thick samples, respectively. The counts in (B) to (D) are normalized to the maximum peak height. The dashed lines in the plots from (B) to (D) are the baseline (which represents the background counts). The baselines were obtained by fitting the data to a sum of Gaussian functions and a baseline *B*. The fitted peaks can be found in Figure S2.

To ascertain that the above assessment from our simulation is accurate, we tested the detector response experimentally by using samples of thickness that varied by three orders of magnitude: 10s of microns, 100s of microns, and 1000 μm. Three resin-embedded particulate Cs-134 samples of different thicknesses (70 ± 40, 680 ± 30, and 1000 ± 200 μm) were measured with the BeaQuant system. Their respective autoradiograph (scatterplot) and spatial distributions for selected regions of interest are displayed in Figure 5.

Based on Figure 5, the difference between the samples was the increase of baseline for increasing sample thickness, while the height and width of the peaks did not change substantially across the different sample thicknesses. These plots reinforce the findings from the GEANT4 simulation: differences in sample thickness (beyond 50 μm) only contribute to higher background and do not affect the counting of the peaks.

One distinct advantage of the BeaQuant system over phosphor screen autoradiography is its ability to store the peak data, in the form of a list of detected events with the exact (*x, y*) reconstructed coordinates and other variables, to perform further quantitative analyses, such as peak fitting. In this study, at least 7 peaks from the magenta plots (in Figure 5B–D) were fitted per sample (see the Supporting Information, Text S2 and Table S2) to obtain the baseline and their individual FWHM. The average FWHMs were 0.6 ± 0.3, 0.9 ± 0.6, and 0.4 ± 0.1 mm for sample thicknesses 70, 680, and 1000 μm, respectively. From the values, each particle must be at least 400 to 900 μm apart to be seen as a distinct particle. Thus, the particles need to be spread out evenly and sparsely (ideally 900 μm from one another), to prevent oversaturation and to obtain accurate quantification of the particles. Data from 20 soil samples surrounding the FDNPP reported that the CsMP’s number density is between 0.869 to 318 particles per gram.^26^ In comparison, the BeaQuant spatial limit of 900 μm per particle would be sufficiently high for the measurement of these samples.

Comparison of the FWHMs among the different samples indicates that the values are similar, which suggests that the peaks observed in the plots were from near the sample surface, regardless of sample thickness. However, we noticed that the FWHMs (0.6 ± 0.3, 0.9 ± 0.6, and 0.4 ± 0.1 mm) were above 182.8 μm, which coincides with the GEANT4 simulated FWHM for a particle beyond the depth of 100 μm from the sample surface, as shown in Table 2. Since the thinnest sample is below 100 μm, it is more probable that the FWHM from the measured samples were for the particles within the first 50 μm of depth but were larger than the simulated FWHM from GEANT4. We speculate that this discrepancy can be attributed to two reasons. First, in the GEANT4 simulation, the particle was assumed to have a 10 μm radius, whereas the particles in the sample have a variation of sizes. For a larger particle, the spread of the peak would be larger. Second, our simulation did not account for the peak broadening effects as the β particles travel through the detector, since we assume the scenario of an ideal detector. Hence, the FWHM calculated by our simulation is an underestimate of the actual values measured by the detector. Future studies could investigate the effect of the detector on the broadening of peaks by artificially introducing gaussian blurring into the GEANT4 simulation.

We also noted the high uncertainty (standard deviation of the peaks) of the FWHM across all three samples (≥25% uncertainty). These high uncertainties suggest that the peaks were indeed originating from particles of varying sizes and within a range of depth. Based on the GEANT4 simulation shown in Figure 4, the ideal sample thickness to study only particles found on the sample surface is 20 μm. However, it is difficult to achieve a 20 μm sample thickness due to unevenness in polishing. For example, in the 70 μm sample, the percentage uncertainty of the thickness is 57% and parts of the sample were already polished off, as seen in Figure S3 and Table S3. The implementation of micro X-Ray CT scans in our study enabled a more accurate GEANT4 simulation of the samples as it provides additional information on the particle’s sizes and spatial *z* coordinates.

**Figure 6:**
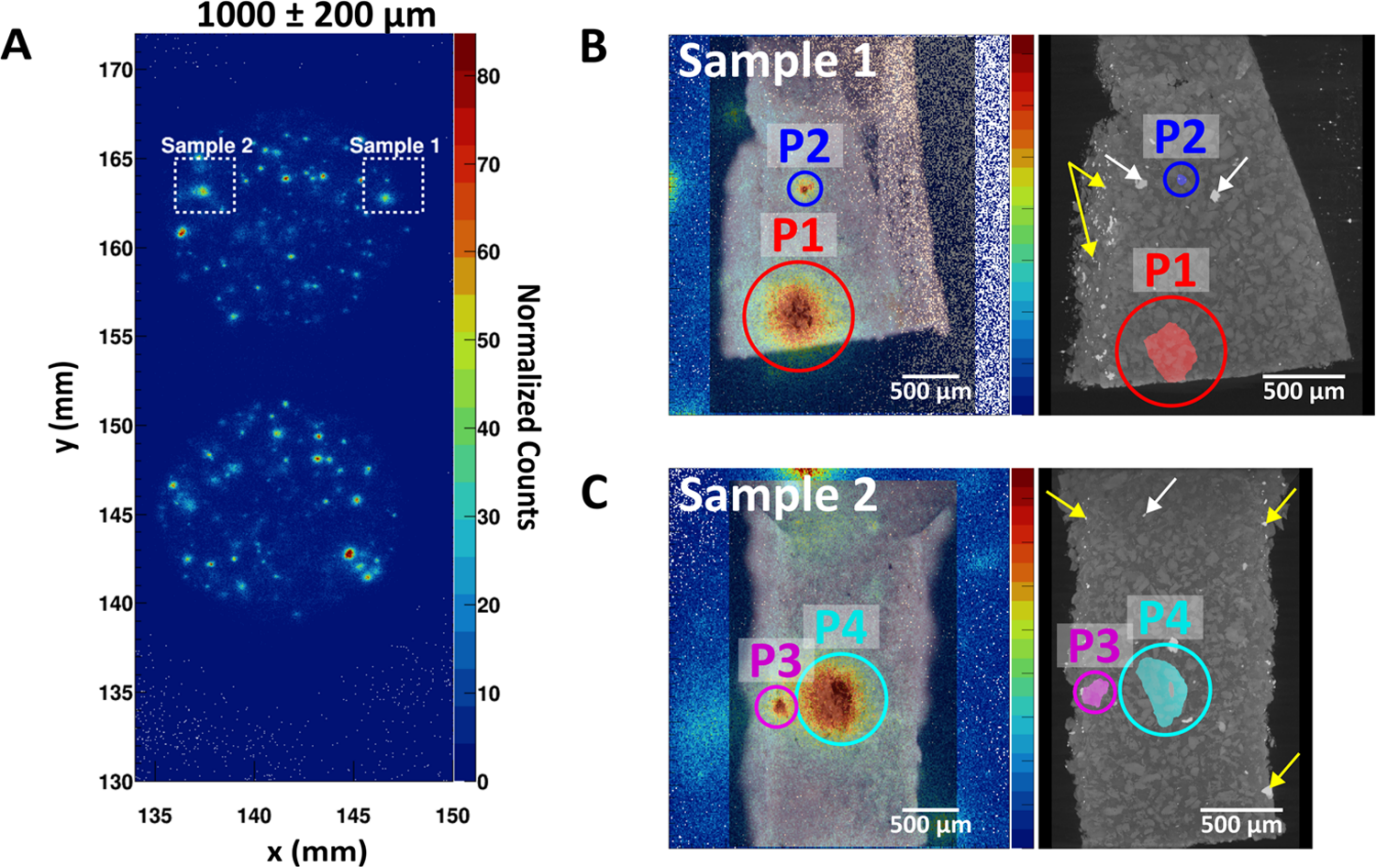
X-ray CT data for 2 regions of interest. (A) Positions of the regions of interest obtained from the autoradiograph of the 1000 μm thick sample. From the region of interest, 2 samples were cut and mounted for micro X-ray CT analysis. The optical microscope images of the samples were superposed to the autoradiograph (left) to identify the radioactive particle positions (highlighted by the circles and particle labels), and the micro X-ray CT 3D projected image is shown beside (right) the autoradiograph for both (B) sample 1 and (C) sample 2. Bright spots found in the edges of the CT scans (yellow arrows) are mostly due to the residual metal from the cutting process with the scalpel, whereas bright spots within the sample (white arrows) can be attributed to the metal contamination after the ball milling of the quartz using stainless steel. Videos showing the 3D projected samples can be found in the Supporting Information (XCT-Movie-S1.avi and XCT-Movie-S2.avi).

Figure 6 shows the 4 particles identified from the 2 regions of interest which were sampled from the 1 mm thick sample. From the analysis, we obtained the size of the particle and its depth from the sample surface. For the simulation, each particle was approximated to an ellipsoid. Table 3 reports the parameters inputted into our simulation based on the information obtained from the micro X-ray CT data.

**Table 3:** Compilation of the Data from the ImageJ Measurements Used in the Ellipsoid Fitting in Our Simulation^a^

particle	distance from surface (μm)	semiaxis A (μm)	semiaxis B (μm)	semiaxis C (μm)
P1	167	140	175	129
P2	28	29	33	28
P3	161	94	63	82
P4	240	126	212	210

a Distance from the surface refers to the *z*-axis distance of the ellipsoid centroid to the sample surface. The particle sizes were estimated with the semiaxes A, B, and C, which refers to the half-axis in the *x*, *y*, and *z* axes, respectively.

As shown in Table 3, the sizes of the particles were larger than the desired size (≤25 μm from the sieving of the Cu-HCF). This could be due to the clumping of the particles during the drying process after Cs-134 sorption onto the Cu-HCF. In the future, we propose suspending the Cu-HCF particles in a surfactant during storage to prevent clumping.

**Figure 7:**
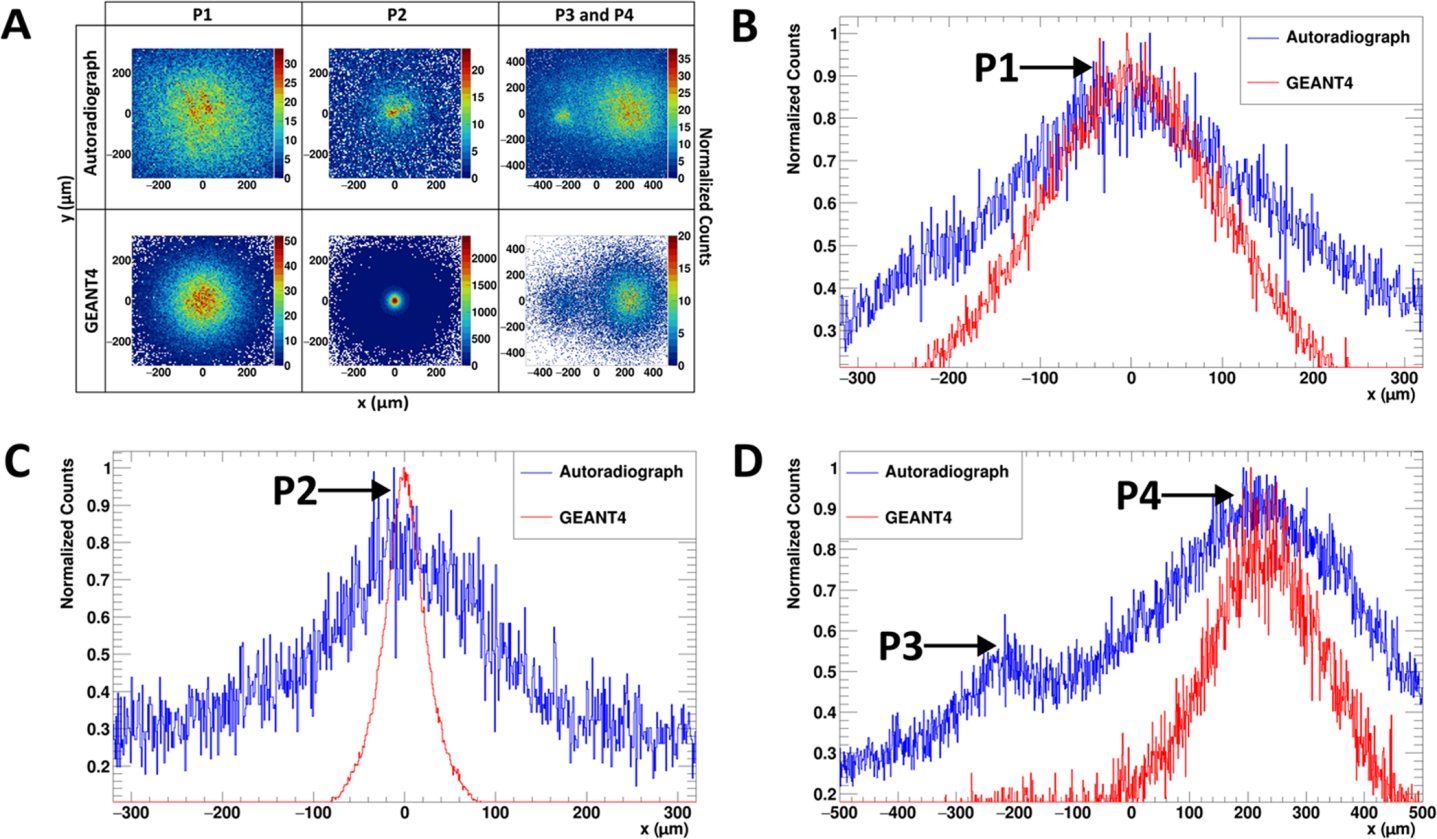
Comparison between simulation and BeaQuant data. (A) Scatterplots (6.4 μm × 6.4 μm per pixel) of the particles from the simulation and the autoradiograph acquired from the BeaQuant system, and the corresponding spatial distribution for particles (B) P1, (C) P2, and (D) P3 and P4. The counts in (B) to (D) are normalized to the maximum peak height. For the simulation in (D), the ratio of intensities from Cs-134 emission between P3 and P4 was estimated by taking the ratio of the particle’s volume (0.109), as calculated with the ImageJ analysis of the CT scan.

Comparison between the simulation data and autoradiograph are presented in Figure 7. We were able to successfully reconstruct the autoradiograph using our simulation. The main difference between our simulation and the autoradiograph is the broadening of the peak and extra background radiation contribution from the autoradiograph. The detector broadening effect can be attributed to the interaction of the electrons (with the gas mixture and micromeshes) within the detector, whereby the trajectory of the electron is random and its subsequent (*x*, *y*) positions after each ionization event would deviate from its vertex (original position at the sample source). These interactions are not accounted for by our simulation; the scatterplots and spatial distributions computed by our simulation represent the position of the electron directly at the sample surface before it enters the detector amplification space. The broadening effect of these interactions could be considered when advancing the simulation in future studies. Figure 7D shows that the BeaQuant system has the ability to distinguish between 2 individual peaks even though P3 (semiaxis A = 94 μm) and P4 (semiaxis A = 126 μm) were 465 μm (centroid-to-centroid distance in the *x*-axis) apart, which suggests that the particles do not have to be too sparsely spread for radioactive particle analysis with the BeaQuant system. In addition, this measured distance is comparable to the FWHM (400 ± 100 μm) for the 1 mm sample measured previously, indicating that our peak fitting was optimal. These findings reinforce that gaseous PIM detectors are a promising tool for the identification of radioactive particles.

### 2.5 Detector Limitations and Implications for Environmental Research

In this study, we have shown that the detector has sufficient sensitivity (in terms of MDA and spatial resolution) for application to environmental samples. However, for the detector to be utilized efficiently, a more refined protocol for sample preparation would be required. For phosphor screen autoradiography, soil samples can be exposed to an imaging plate without much modification (e.g., sieving, drying, etc.). In contrast, with our particulate standards, resin embedding was used. This technique takes a few days of sample preparation (curing of the resin, sawing, and polishing). Introducing a more reversible and faster way to ready samples for detection (e.g., setting the sample with an agar medium) is a next step for this research.

## 3 Conclusions

We have proposed and demonstrated the use of thin-layer samples to achieve optimal detector calibration and efficiency calculations suitable for radiation surface contamination studies. The intrinsic efficiency obtained for Cs-134 and Cs-137 would serve as a benchmark for future radio-Cs measurements in the environment. Using the same samples, we calculated the MDA, which will advise BeaQuant system users on the detection limits of radio-Cs. From the MDA, we suggest that the BeaQuant system is sensitive enough to detect radio-Cs found in environmental samples, such as CsMPs originating from the FDNPP accident. Moreover, the particles could be detected (with sufficient signal for analysis) after less than a day of acquisition (>16 h). Digital autoradiography with a gaseous detector has a time advantage over the conventional technique of phosphor screen autoradiography, which will expedite the monitoring and detection of radioactive particles during time-sensitive scenarios (i.e., emergency/accident response). However, current sample preparation techniques would need to be improved to maximize the advantage of rapid detection. A high correlation between the GEANT4 simulations and experimental results proved that the simulation model is successful in understanding the detector function response. While the work presented here is directed toward the detection and quantification of Cs radioisotopes, our simulation and detector optimization techniques could be applied to other radionuclides. While the experimental data revealed that we are unable to separate Cs-134 and Cs-137 via deconvolution, the divergence in energy deposition displayed by the GEANT4 simulation appears promising. Future advancement in the detector’s electronics might facilitate the ability to separate these radioisotopes. Despite the detector’s inability to separate Cs-134 and Cs-137 via deconvolution, at present, our method of detection can be used as a tool to select and extract CsMPs, before subjecting the particle to further characterization (e.g., isotopic ratio determination with γ spectroscopy). A new class of resin-embedded particulate standards, which simulated environmental samples, were produced. These samples enabled an in-depth study of the effect of sample thickness on signal quality. Results revealed that samples beyond the thickness of 50 μm do not detrimentally affect the signal. Our work on the BeaQuant system, an example of MPGD incorporating a PIM structure, highlighted the potential and importance of MPGD application toward rapid radiation detection in environmental samples. Overall, we have generated a framework to detect radio-Cs found in the environment using a detector based on PIM gaseous detectors.

## 4 Materials and Methods

### 4.1 Preparation of Thin-Layer Samples

Thin-layer samples were made to be virtually “massless” to maximize spectral resolution. These samples were deposited by aliquoting 10 μL of radio-Cs solution, via pipette, onto a glass slide, and the solution was then evaporated to dryness. Several solutions were prepared using serial dilutions to obtain seven different activity concentrations of Cs-134 and Cs-137, respectively. These samples served as calibration standards for the MPGD (Figure S1). Results from the calibration curve were used to calculate the absolute efficiency ϵ_abs_ by taking the ratio of the measured count rates (acquired from the BeaQuant system) to the samples’ known activities (determined from the γ spectroscopy measurements). Another set of solutions containing varying Cs-134: Cs-137 activity concentration ratios (1:0; 0.69:0.31; 0.40:0.60; 0.17:0.83; and 0:1) were prepared. Cellulose nanocrystals (0.1 w/v%) were added to all solutions to improve the quality of the resulting dried samples, by minimizing the “coffee-ring” effect.^45^ The resulting samples had activity ranges from ∼1 to 100 Bq, which were measured using γ spectroscopy. The γ measurements (acquisition time between 1.3 to 165 h for good counting statistics of ≤1% uncertainty) were conducted using a Canberra GC4018 detector (Coaxial HPGe Detector) which has a resolution of 1.8 keV at 1.33 MeV. Cs-134 was measured from the 604.7 keV γ peak (97.62% intensity) and Cs-137 from the 661.7 keV peak (85.10% intensity). Each spectrum was analyzed with the Genie 2000 Gamma Acquisition & Analysis software. The effect of the measurement geometry on γ efficiency was determined using an Eckert & Ziegler calibration standard solution.

After drying, the samples were wrapped with a layer of 3 μm mylar film (Chemplex Industries) to prevent contamination of the micromesh of the MPGD during further analysis. An example image of a sample is shown in Figure S1. In addition to the thin-layer samples, blank samples (without any radioactivity) were prepared in the same way as detailed above, to assess the background and obtain the detection limits of the MPGD.

### 4.2 Preparation of Cs-134 Labeled Particles

Copper hexacyanoferrate (Cu-HCF) microparticles were used as a simulant (size, morphology, possible Cs-concentrations) for CsMPs due to the high adsorption capacity of Cu-HCF for Cs and its good chemical stability across a wide pH range.^46,47^ The synthesis of Cu-HCF was adopted from Harjula et al.^48^ 50 mL of 0.65 M K_4_Fe(CN)_6_ solution was slowly poured into 80 mL of 1 M CuNO_3_ solution, which was vigorously stirred by a magnetic stirrer. The slurry obtained from the reaction was left in the mixture for 30 minutes before it was washed with Milli-Q water. The washed material was dried in an open atmosphere oven at 70 °C overnight. Prior to Cs-134 adsorption, Cu-HCF was ground with a mortar and pestle and sieved to a size fraction of <25 μm. Cs-134 was sorbed (Figure S4) onto the particles to match the activity per CsMP found in environmental samples (>0.06 Bq per particle for particles less than 114 μm).^20^

After sorption, the Cs-134 labeled Cu-HCF particles were air-dried and mixed with acid-washed nonradioactive quartz (size fraction 50–100 μm). To ensure that the samples were fixed in place, the mixture of particles and quartz was resin-embedded using epoxy resin Araldite M (1.038 g/cm^3^ at 25 °C) and hardener REN HY956 (1.02 g/cm^3^ at 25 °C) with a mass ratio of 5:1. The resin was cured overnight before being sawn to 1 mm thickness. The resulting section was adhered onto a glass slide using a thin film of the same resin and hardener. The samples were subsequently polished with polishing diamond plates of grit size 80, 500, 1200, and 2000, respectively (MD-Piano, Struers). The samples were polished down to three different sample thickness ranges (tens of μm, hundreds of μm, and 1 mm). The final thickness of the samples was quantified using either the polarizing microscope (thickness < 100 μm) or a micrometer dial indicator (thickness > 100 μm). Images of the samples and their measured thicknesses are shown in Figure S3 and Table S3.

### 4.3 Real-Time Digital Autoradiography Using MPGD with the BeaQuant System

Autoradiographs were acquired using a BeaQuant, a commercially available MPGD incorporating micromesh PIM that facilitates real-time autoradiography.^33^ Before the samples were loaded into the detector, compressed air was used to carefully remove any dust or impurities from the samples.

To ensure good counting statistics, the acquisition time for samples used in this study varied between 17 to 66 h. In this work, the dead time contribution from the detector is insignificant due to the low radioactivity of the samples used. The resulting data were analyzed with the software Beamage (version 3.3) and CERN ROOT (version 6.19/02) for image reconstruction.^49^

### 4.4 Micro X-ray Computed Tomography Scans

The Cs-134 particle-containing samples were visualized in three dimensions (3D) using micro X-ray computed tomography (Xradia MicroXCT-400). X-ray CT provides the positions of the radioactive particles in the *z*-axis. The 1 mm thick resin-embedded sample was cut to approximately 1–2 mm in length and width (using a saw and scalpel) to be scanned by the X-ray CT. Prior to imaging, the samples were mounted onto a carbon rod with epoxy resin (Casco Strong Epoxy SuperQuick). The samples were imaged using microfocus X-ray source parameters of 40 kV and 100 μA without any filter. A 10× microscope objective lens (equipped with a scintillator) was used together with camera binning mode 2, in which all 2 × 2 pixels were combined to work as a single pixel. The resulting image pixel size was 2.33 μm. A total of 571 projection images (exposure time 2.5 s per projection) were taken over a 190° rotation using a 0.33° step size. Finally, the projection images were reconstructed into tomographic slices using the filtered backprojection algorithm.^50^ To locate the particles, Inkscape was used to superpose the images obtained from autoradiography, optical microscopy (Leica Z16 APO), and the acquired X-ray CT scans. Subsequently, ImageJ software was used for the analysis of the X-ray CT images to obtain their spatial information.^51^ Four Cs-134-containing particles (in two samples) were identified and measured with this technique. The information was used in simulations for comparison to the autoradiograph.

### 4.5 Monte Carlo Simulations

Monte Carlo simulations were carried out using GEANT4 (GEometry ANd Tracking 4, version 4.10.07), a C++ toolkit that enables the simulation of charged particles, γ-rays, and optical photons’ transport and interaction through matter.^52^ GEANT4 models of the two types of samples (thin-layer and resin-embedded Cs-134 particles) were created. The geometrical visualization of the two models is presented in Figure S5. In the thin-layer sample, GEANT4 simulations were run for both Cs-134 and Cs-137 to observe the difference in the electron energy deposition into the detector. In addition, GEANT4 was also used to obtain the fraction of total electrons entering the detector from the sample surface over the total electrons emitted from the sample through radiation decay, referred to as *F*_e_, using eq 1. For particle-containing samples, GEANT4 has been used to study the β particle distributions from a spherical Cs-134 containing particle (with 10 μm radius) placed at varying depths (0, 10, 20, 30, 40, 50, 100, 500 μm, and 1 mm) from the sample surface. One million decay events were set for each run. The energy distribution of electrons, energy deposited in amplification space 1 (see Figure 1), and spatial distribution of the electrons, were recorded and plotted in histograms using ROOT environment.^49^

## Supporting Information Available

Formulas for critical level, detection limit, and minimum detectable activity (Text S1); peak fitting formulation (Text S2); calibration of the thin-layer sample (Figure S1); peak fitting for BeaQuant data with Cs-134 particle samples of varying thicknesses (Figure S2); images of resin-embedded samples with Cs-134 particles mixed with nonradioactive quartz (Figure S3); adsorption rate plot of Cs onto Cu-HCF (Figure S4); geometrical visualization of the Monte Carlo simulation with GEANT4 (Figure S5); energies and intensities of the electrons emitted by Cs-134 and Cs-137 (Table S1); summary of fitting parameters and FWHM for each peak from Figure S2 (Table S2); and measured thickness of the resin-embedded samples (Table S3) (PDF)
Micro X-ray CT 3D projected video of sample 1 (AVI)
Micro X-ray CT 3D projected video of sample 2 (AVI)

## Supplementary Material

ao3c00728_si_001.pdf

ao3c00728_si_002.avi

ao3c00728_si_003.avi

## References

[ref1] Doi T.; Masumoto K.; Toyoda A.; Tanaka A.; Shibata Y.; Hirose K. Anthropogenic Radionuclides in the Atmosphere Observed at Tsukuba: Characteristics of the Radionuclides Derived from Fukushima. J. Environ. Radioact. 2013, 122, 55–62. 10.1016/J.JENVRAD.2013.02.001. 23542231

[ref2] le Petit G.; Douysset G.; Ducros G.; Gross P.; Achim P.; Monfort M.; Raymond P.; Pontillon Y.; Jutier C.; Blanchard X.; Taffary T.; Moulin C. Analysis of Radionuclide Releases from the Fukushima Dai-Ichi Nuclear Power Plant Accident Part I. Pure Appl. Geophys. 2014, 171, 629–644. 10.1007/s00024-012-0581-6.

[ref3] de Cort M.; Dubois G.; Fridman S. D.; Germenchuk M. G.; Izrael Y. A.; Janssens A.; Jones A. R.; Kelly G. N.; Kvasnikova Ev.; Matveenko I. I.; Nazarov I. M.; Pokumeiko Y. M.; Sitak V. A.; Stukin E. D.; Tabachny L. Y.; Tsaturov Y. S.; Avdyushin S. I. Atlas of Caesium Deposition on Europe after the Chernobyl Accident; Luxembourg, 1998.

[ref4] Furuki G.; Imoto J.; Ochiai A.; Yamasaki S.; Nanba K.; Ohnuki T.; Grambow B.; Ewing R. C.; Utsunomiya S. Caesium-Rich Micro-Particles: A Window into the Meltdown Events at the Fukushima Daiichi Nuclear Power Plant. Sci. Rep. 2017, 7, 42731 10.1038/srep42731. 28198440 PMC5309886

[ref5] Yamaguchi N.; Mitome M.; Kotone A.-H.; Asano M.; Adachi K.; Kogure T. Internal Structure of Cesium-Bearing Radioactive Microparticles Released from Fukushima Nuclear Power Plant. Sci. Rep. 2016, 6, 20548 10.1038/srep20548. 26838055 PMC4738348

[ref6] Ohnuki T.; Satou Y.; Utsunomiya S. Formation of Radioactive Cesium Microparticles Originating from the Fukushima Daiichi Nuclear Power Plant Accident: Characteristics and Perspectives. J. Nucl. Sci. Technol. 2019, 56, 790–800. 10.1080/00223131.2019.1595767.

[ref7] Wakiyama Y.; Konoplev A.; Wada T.; Takase T.; Byrnes I.; Carradine M.; Nanba K. Behavior of ^137^Cs in Ponds in the Vicinity of the Fukushima Dai-Ichi Nuclear Power Plant. J. Environ. Radioact. 2017, 178–179, 367–376. 10.1016/J.JENVRAD.2017.07.017. 28797551

[ref8] Kaeriyama H. Oceanic Dispersion of Fukushima-Derived Radioactive Cesium: A Review. Fish. Oceanogr. 2017, 26, 99–113. 10.1111/fog.12177.

[ref9] International Atomic Energy Agency. Environmental Consequences of the Chernobyl Accident and Their Remediation: Twenty Years of Experience; Vienna, 2006.

[ref10] Ministry of the Environment. BOOKLET to provide basic information regarding health effects of radiation. https://www.env.go.jp/en/chemi/rhm/basic-info/1st/02-02-05.html (accessed Dec 08, 2022).

[ref11] Povinec P. P.; Hirose K.; Aoyama M. Radionuclide Releases into the Environment. In Fukushima Accident; Elsevier, 2013; pp 103–130.

[ref12] Chino M.; Nakayama H.; Nagai H.; Terada H.; Katata G.; Yamazawa H. Preliminary Estimation of Release Amounts of ^131^I and ^137^Cs Accidentally Discharged from the Fukushima Daiichi Nuclear Power Plant into the Atmosphere. J. Nucl. Sci. Technol. 2011, 48, 1129–1134. 10.1080/18811248.2011.9711799.

[ref13] Morino Y.; Ohara T.; Nishizawa M. Atmospheric Behavior, Deposition, and Budget of Radioactive Materials from the Fukushima Daiichi Nuclear Power Plant in March 2011. Geophys. Res. Lett. 2011, 38, L00G11 10.1029/2011GL048689.

[ref14] Tanaka K.; Takahashi Y.; Sakaguchi A.; Umeo M.; Hayakawa S.; Tanida H.; Saito T.; Kanai Y. Vertical Profiles of Iodine-131 and Cesium-137 in Soils in Fukushima Prefecture Related to the Fukushima Daiichi Nuclear Power Station Accident. Geochem. J. 2012, 46, 73–76. 10.2343/geochemj.1.0137.

[ref15] Kozai N.; Ohnuki T.; Arisaka M.; Watanabe M.; Sakamoto F.; Yamasaki S.; Jiang M. Chemical States of Fallout Radioactive Cs in the Soils Deposited at Fukushima Daiichi Nuclear Power Plant Accident. J. Nucl. Sci. Technol. 2012, 49, 473–478. 10.1080/00223131.2012.677131.

[ref16] Matsunami H.; Murakami T.; Fujiwara H.; Shinano T. Evaluation of the Cause of Unexplained Radiocaesium Contamination of Brown Rice in Fukushima in 2013 Using Autoradiography and Gamma-Ray Spectrometry. Sci. Rep. 2016, 6, 20386 10.1038/srep20386. 26843000 PMC4740882

[ref17] Burger A.; Lichtscheidl I. Stable and Radioactive Cesium: A Review about Distribution in the Environment, Uptake and Translocation in Plants, Plant Reactions and Plants′ Potential for Bioremediation. Sci. Total Environ. 2018, 618, 1459–1485. 10.1016/j.scitotenv.2017.09.298. 29122347

[ref18] Connor D. T.; Martin P. G.; Smith N. T.; Payne L.; Hutson C.; Payton O. D.; Yamashiki Y.; Scott T. B. Application of Airborne Photogrammetry for the Visualisation and Assessment of Contamination Migration Arising from a Fukushima Waste Storage Facility. Environ. Pollut. 2018, 234, 610–619. 10.1016/j.envpol.2017.10.098. 29223818

[ref19] Satou Y.; Sueki K.; Sasa K.; Adachi K.; Igarashi Y. First Successful Isolation of Radioactive Particles from Soil near the Fukushima Daiichi Nuclear Power Plant. Anthropocene 2016, 14, 71–76. 10.1016/j.ancene.2016.05.001.

[ref20] Ikehara R.; Suetake M.; Komiya T.; Furuki G.; Ochiai A.; Yamasaki S.; Bower W. R.; Law G. T. W.; Ohnuki T.; Grambow B.; Ewing R. C.; Utsunomiya S. Novel Method of Quantifying Radioactive Cesium-Rich Microparticles (CsMPs) in the Environment from the Fukushima Daiichi Nuclear Power Plant. Environ. Sci. Technol. 2018, 52, 6390–6398. 10.1021/acs.est.7b06693. 29782160

[ref21] Suetake M.; Nakano Y.; Furuki G.; Ikehara R.; Komiya T.; Kurihara E.; Morooka K.; Yamasaki S.; Ohnuki T.; Horie K.; Takehara M.; Law G. T. W.; Bower W.; Grambow B.; Ewing R. C.; Utsunomiya S. Dissolution of Radioactive, Cesium-Rich Microparticles Released from the Fukushima Daiichi Nuclear Power Plant in Simulated Lung Fluid, Pure-Water, and Seawater. Chemosphere 2019, 233, 633–644. 10.1016/j.chemosphere.2019.05.248. 31195267

[ref22] Adachi K.; Kajino M.; Zaizen Y.; Igarashi Y. Emission of Spherical Cesium-Bearing Particles from an Early Stage of the Fukushima Nuclear Accident. Sci. Rep. 2013, 3, 2554 10.1038/srep02554. 23989894 PMC3757362

[ref23] Morooka K.; Kurihara E.; Takehara M.; Takami R.; Fueda K.; Horie K.; Takehara M.; Yamasaki S.; Ohnuki T.; Grambow B.; Law G. T. W.; Ang J. W. L.; Bower W. R.; Parker J.; Ewing R. C.; Utsunomiya S. New Highly Radioactive Particles Derived from Fukushima Daiichi Reactor Unit 1: Properties and Environmental Impacts. Sci. Total Environ. 2021, 773, 145639 10.1016/j.scitotenv.2021.145639. 33940743

[ref24] International Atomic Energy Agency. Environmental Behaviour and Potential Biological Impact of Radioactive Particles. CRP1944 Project Code K41013. IAEA October 12 2018.

[ref25] Matsuya Y.; Hamada N.; Yachi Y.; Satou Y.; Ishikawa M.; Date H.; Sato T. Inflammatory Signaling and DNA Damage Responses after Local Exposure to an Insoluble Radioactive Microparticle. Cancers 2022, 14, 1045 10.3390/cancers14041045. 35205797 PMC8869995

[ref26] Ikehara R.; Morooka K.; Suetake M.; Komiya T.; Kurihara E.; Takehara M.; Takami R.; Kino C.; Horie K.; Takehara M.; Yamasaki S.; Ohnuki T.; Law G. T. W.; Bower W.; Grambow B.; Ewing R. C.; Utsunomiya S. Abundance and Distribution of Radioactive Cesium-Rich Microparticles Released from the Fukushima Daiichi Nuclear Power Plant into the Environment. Chemosphere 2020, 241, 125019 10.1016/j.chemosphere.2019.125019. 31610456

[ref27] Abe Y.; Onozaki S.; Nakai I.; Adachi K.; Igarashi Y.; Oura Y.; Ebihara M.; Miyasaka T.; Nakamura H.; Sueki K.; Tsuruta H.; Moriguchi Y. Widespread Distribution of Radiocesium-Bearing Microparticles over the Greater Kanto Region Resulting from the Fukushima Nuclear Accident. Prog. Earth Planet Sci. 2021, 8, 13 10.1186/s40645-020-00403-6.

[ref28] Utsunomiya S.; Furuki G.; Ochiai A.; Yamasaki S.; Nanba K.; Grambow B.; Ewing R. C. Caesium Fallout in Tokyo on 15th March, 2011 Is Dominated by Highly Radioactive, Caesium-Rich Microparticles. 2019, arXiv: 1906.00212v2. arXiv.org e-Print archive. https://arxiv.org/abs/1906.00212.

[ref29] Miura H.; Kurihara Y.; Sakaguchi A.; Tanaka K.; Yamaguchi N.; Higaki S.; Takahashi Y. Discovery of Radiocesium-Bearing Microparticles in River Water and Their Influence on the Solid-Water Distribution Coefficient (K_d_) of Radiocesium in the Kuchibuto River in Fukushima. Geochem. J. 2018, 52, 145–154. 10.2343/geochemj.2.0517.

[ref30] Miura H.; Ishimaru T.; Ito Y.; Kurihara Y.; Otosaka S.; Sakaguchi A.; Misumi K.; Tsumune D.; Kubo A.; Higaki S.; Kanda J.; Takahashi Y. First Isolation and Analysis of Caesium-Bearing Microparticles from Marine Samples in the Pacific Coastal Area near Fukushima Prefecture. Sci. Rep. 2021, 11, 5664 10.1038/s41598-021-85085-w. 33707572 PMC7952385

[ref31] Sanada Y.; Urabe Y.; Misonou T.; Shiribiki T.; Nakanishi T.; Watanabe Y.; Tsuruta T. Visualization of Radiocesium Distribution in Surface Layer of Seafloor around Fukushima Daiichi Nuclear Power Plant. Sci. Rep. 2021, 11, 23175 10.1038/s41598-021-02646-9. 34848808 PMC8633336

[ref32] Nishizawa Y.; Yoshida M.; Sanada Y.; Torii T. Distribution of the ^134^Cs/^137^Cs Ratio around the Fukushima Daiichi Nuclear Power Plant Using an Unmanned Helicopter Radiation Monitoring System. J. Nucl. Sci. Technol. 2016, 53, 468–474. 10.1080/00223131.2015.1071721.

[ref33] Donnard J.; Berny R.; Carduner H.; Leray P.; Morteau E.; Provence M.; Servagent N.; Thers D. The Micro-Pattern Gas Detector PIM: A Multi-Modality Solution for Novel Investigations in Functional Imaging. Nucl. Instrum. Methods Phys. Res., Sect. A 2009, 610, 158–160. 10.1016/j.nima.2009.05.186.

[ref34] Thers D.; Bretonniere T.; Charpak G.; Coulon P.; Leray P.; Drancourt C.; le Guay M.; Lupone S.; Luquin L.; Martínez G.; Meynadier M.; Pichot P. Parallel Ionization Multiplier (PIM): A New Concept of Gaseous Detector for Radiation Detection Improvement. Nucl. Instrum. Methods Phys. Res., Sect. A 2003, 504, 161–165. 10.1016/S0168-9002(03)00813-1.

[ref35] Delayre C.; Sammaljärvi J.; Billon S.; Muuri E.; Sardini P.; Siitari-Kauppi M. Comparison of Phosphor Screen Autoradiography and Micro-Pattern Gas Detector Based Autoradiography for the Porosity of Altered Rocks. Sci. Rep. 2020, 10, 9455 10.1038/s41598-020-65791-7. 32528033 PMC7289799

[ref36] Lefeuvre H.; Donnard J.; Descostes M.; Billon S.; Duval S.; Oger T.; Toubon H.; Sardini P. Spectroscopic Autoradiography of Alpha Particles Using a Parallel Ionization Multiplier Gaseous Detector. Nucl. Instrum. Methods Phys. Res., Sect. A 2022, 1035, 166807 10.1016/j.nima.2022.166807.

[ref37] Billon S.; Sardini P.; Leblond S.; Fichet P. From Bq Cm^–3^ to Bq Cm^–2^ (and Conversely)—Part 1: A Useful Conversion for Autoradiography. J. Radioanal. Nucl. Chem. 2019, 320, 643–654. 10.1007/s10967-019-06521-w.

[ref38] Muuri E.; Sorokina T.; Donnard J.; Billon S.; Helariutta K.; Koskinen L.; Martin A.; Siitari-Kauppi M. Electronic Autoradiography of ^133^Ba Particle Emissions; Diffusion Profiles in Granitic Rocks. Appl. Radiat. Isot. 2019, 149, 108–113. 10.1016/j.apradiso.2019.04.026. 31048201

[ref39] Currie L. A. Limits for Qualitative Detection and Quantitative Determination Application to Radiochemistry. Anal. Chem. 1968, 40, 586–593. 10.1021/ac60259a007.

[ref40] National Nuclear Data Center at Brookhaven National Laboratory. NuDat 3.0. https://www.nndc.bnl.gov/nudat3/ (accessed Aug 09, 2022).

[ref41] Endo S.; Tanaka K.; Kajimoto T.; Tat Thanh N.; Otaki J. M.; Imanaka T. Estimation of β-Ray Dose in Air and Soil from Fukushima Daiichi Power Plant Accident. J. Radiat. Res. 2014, 55, 476–483. 10.1093/jrr/rrt209. 24504671 PMC4014171

[ref42] Talaat K.; Xi J.; Baldez P.; Hecht A. Radiation Dosimetry of Inhaled Radioactive Aerosols: CFPD and MCNP Transport Simulations of Radionuclides in the Lung. Sci. Rep. 2019, 9, 17450 10.1038/s41598-019-54040-1. 31768010 PMC6877642

[ref43] Donnard J. Étude et Conception d′un Imageur Bêta à Très Haute Résolution Spatiale; Université de Nantes: Nantes, 2008. https://tel.archives-ouvertes.fr/tel-00769792.

[ref44] Ochiai A.; Imoto J.; Suetake M.; Komiya T.; Furuki G.; Ikehara R.; Yamasaki S.; Law G. T. W.; Ohnuki T.; Grambow B.; Ewing R. C.; Utsunomiya S. Uranium Dioxides and Debris Fragments Released to the Environment with Cesium-Rich Microparticles from the Fukushima Daiichi Nuclear Power Plant. Environ. Sci. Technol. 2018, 52, 2586–2594. 10.1021/acs.est.7b06309. 29378406

[ref45] Ooi Y.; Hanasaki I.; Mizumura D.; Matsuda Y. Suppressing the Coffee-Ring Effect of Colloidal Droplets by Dispersed Cellulose Nanofibers. Sci. Technol. Adv. Mater. 2017, 18, 316–324. 10.1080/14686996.2017.1314776. 28567177 PMC5439399

[ref46] Lee K.-M.; Kawamoto T.; Minami K.; Takahashi A.; Parajuli D.; Kido G.; Yoshino K.; Tanaka H. Improved Adsorption Properties of Granulated Copper Hexacyanoferrate with Multi-Scale Porous Networks. RSC Adv. 2016, 6, 16234–16238. 10.1039/c5ra25388h.

[ref47] Nilchi A.; Saberi R.; Moradi M.; Azizpour H.; Zarghami R. Adsorption of Cesium on Copper Hexacyanoferrate–PAN Composite Ion Exchanger from Aqueous Solution. Chem. Eng. J. 2011, 172, 572–580. 10.1016/j.cej.2011.06.011.

[ref48] Harjula R.; Lehto J. Method for Cesium Removal from Radioactive Waste Liquids and Method for Producing Hexacyanoferrates. EP0909447, December 31, 1997.

[ref49] Brun R.; Rademakers F. ROOT — An Object Oriented Data Analysis Framework. Nucl. Instrum. Methods Phys. Res., Sect. A 1997, 389, 81–86. 10.1016/S0168-9002(97)00048-X.

[ref50] Feldkamp L. A.; Davis L. C.; Kress J. W. Practical Cone-Beam Algorithm. J. Opt. Soc. Am. A 1984, 1, 612–619. 10.1364/JOSAA.1.000612.

[ref51] Schneider C. A.; Rasband W. S.; Eliceiri K. W. NIH Image to ImageJ: 25 Years of Image Analysis. Nat. Methods 2012, 9, 671–675. 10.1038/nmeth.2089. 22930834 PMC5554542

[ref52] Agostinelli S.; Allison J.; Amako K.; Apostolakis J.; Araujo H.; Arce P.; Asai M.; Axen D.; Banerjee S.; Barrand G.; Behner F.; Bellagamba L.; Boudreau J.; Broglia L.; Brunengo A.; Burkhardt H.; Chauvie S.; Chuma J.; Chytracek R.; Cooperman G.; Cosmo G.; Degtyarenko P.; Dell’Acqua A.; Depaola G.; Dietrich D.; Enami R.; Feliciello A.; Ferguson C.; Fesefeldt H.; Folger G.; Foppiano F.; Forti A.; Garelli S.; Giani S.; Giannitrapani R.; Gibin D.; Gómez Cadenas J. J.; González I.; Gracia Abril G.; Greeniaus G.; Greiner W.; Grichine V.; Grossheim A.; Guatelli S.; Gumplinger P.; Hamatsu R.; Hashimoto K.; Hasui H.; Heikkinen A.; Howard A.; Ivanchenko V.; Johnson A.; Jones F. W.; Kallenbach J.; Kanaya N.; Kawabata M.; Kawabata Y.; Kawaguti M.; Kelner S.; Kent P.; Kimura A.; Kodama T.; Kokoulin R.; Kossov M.; Kurashige H.; Lamanna E.; Lampén T.; Lara V.; Lefebure V.; Lei F.; Liendl M.; Lockman W.; Longo F.; Magni S.; Maire M.; Medernach E.; Minamimoto K.; Mora de Freitas P.; Morita Y.; Murakami K.; Nagamatu M.; Nartallo R.; Nieminen P.; Nishimura T.; Ohtsubo K.; Okamura M.; O’Neale S.; Oohata Y.; Paech K.; Perl J.; Pfeiffer A.; Pia M. G.; Ranjard F.; Rybin A.; Sadilov S.; di Salvo E.; Santin G.; Sasaki T.; Savvas N.; Sawada Y.; Scherer S.; Sei S.; Sirotenko V.; Smith D.; Starkov N.; Stoecker H.; Sulkimo J.; Takahata M.; Tanaka S.; Tcherniaev E.; Safai Tehrani E.; Tropeano M.; Truscott P.; Uno H.; Urban L.; Urban P.; Verderi M.; Walkden A.; Wander W.; Weber H.; Wellisch J. P.; Wenaus T.; Williams D. C.; Wright D.; Yamada T.; Yoshida H.; Zschiesche D. GEANT4—a Simulation Toolkit. Nucl. Instrum. Methods Phys. Res., Sect. A 2003, 506, 250–303. 10.1016/S0168-9002(03)01368-8.

