# Supplementary material for: Improved Radio-Cesium Detection Using Quantitative Real-Time Autoradiography
Source: ACS Omega. 2023 Jun 13;8(25):22523–35. doi: 10.1021/acsomega.3c00728 (PMC10308591; doi:10.1021/acsomega.3c00728)
Supplement: Supplementary file 1 — ao3c00728_si_001.pdf [file ao3c00728_si_001.pdf]

Supporting Information for:

Improved Radio-cesium Detection using Quantitative Real-time Autoradiography

Joyce W. L. Ang[a,b,*], Arthur Bongrand[c,d], Samuel Duval[c], Jérôme Donnard[c], Joni Parkkonen[e], Satoshi Utsunomiya[f], Risto Koivula[a], Marja Siitari-Kauppi[a], Gareth T. W. Law[a,*]

[a]Radiochemistry Unit, Department of Chemistry, The University of Helsinki, Helsinki 00014, Finland

[b]Singapore Nuclear Safety and Research Initiative, National University of Singapore, 138602, Singapore

[c]AI4R, 2 rue Alfred Kastler, 44307, Nantes, France

[d]IMT Atlantique, Nantes Université, CNRS, SUBATECH, F-44000 Nantes, France

[e]Department of Physics, University of Jyväskylä, Jyväskylä 40500, Finland

[f]Department of Chemistry, Kyushu University, 744 Motooka, Nishi-ku, Fukuoka 819-0395, Japan

*Email: joyce.ang@helsinki.fi, gareth.law@helsinki.fi

This PDF file includes:

Supplementary Text S1 to S2

Figs. S1 to S5

Tables S1 to S3

Other Supplementary Materials for this manuscript include the following:

Movies S1 to S2

Table of Contents

Text S1: Critical level, detection limit and minimum detectable activity formulae S3

Text S2: Peak fitting formulation S5

Figure S1: Calibration of the thin layer sample S6

Figure S2: Peak fitting for BeaQuant data with Cs-134 particle samples of varying thicknesses S7

Figure S3: Resin embedded sample images S8

Figure S4: Adsorption rate plot of Cs onto Cu-HCF S9

Figure S5: Geometrical visualization of the Monte Carlo simulation with GEANT4 .... S10

Table S1: Energies and intensities of electrons emitted by Cs-134 and Cs-137 .... S11

Table S2: Summary of fitting parameters and FWHM for each peak S12

Table S3: Measured thickness of the resin embedded samples S13

Text S1: Critical level, detection limit and minimum detectable activity formulae

In autoradiography, the spatial area of the background taken would most likely be different from the area of the sample. It is ideal for the background area to be as large as possible to obtain better counting statistics. As a result, normalization of the counts to the area will be the best approach to compute the critical level, detection limit, and minimum detectable activity. However, due to the normalization, the uncertainty of background counts due to statistical fluctuation can no longer be $\sigma_{N_B} = \sqrt{N_B}$. Hence, we cannot use the Currie equation directly,

$$N_D = 4.653\sqrt{N_B} + 2.706 \tag{S1}$$

where N_D is the detection limit, N_B is the background counts, and the probability for type I and type II errors were set at 5%. Here, we elucidate the derivations for L_C, N_D, and MDA in the case of normalized area.

Firstly, we define N_B as the number of recorded counts per mm^2 when a blank sample is loaded into the detector and N_T as the number of recorded counts per mm^2 when measuring a sample that is known to emit some level of radiation. For simplicity, we assume that the acquisition time for the blank and the sample are kept the same. Therefore, the net counts per mm^2 N_s from the sample can be written as:

$$N_S = N_T - N_B\ . \tag{S2}$$

The uncertainty of N_S is given by,

$$\sigma_{N_S} = \sqrt{{\sigma_{N_T}}^2 + {\sigma_{N_B}}^2} \tag{S3}$$

where σ_{N_T} and σ_{N_B} are the uncertainties of N_T and N_B respectively. However, due to the definition of N_B (as counts per 1 mm^2 as opposed to total background counts), for a total background measurement area of A mm^2, the uncertainty of background counts is $\sigma_{N_B} = \frac{\sqrt{N_B \cdot A}}{A} = \sqrt{\frac{N_B}{A}}$ instead of $\sigma_{N_B} = \sqrt{N_B}$.

Considering the case when there are no real activity present, the net counts per mm^2 is $N_S = 0$ and $\sigma_{N_T} = \sigma_{N_B}$. As a result, equation (S3) can be re-written as:

$$\sigma_{N_S} = \sqrt{2 \cdot {\sigma_{N_B}}^2} = \sqrt{\frac{2}{A} \cdot N_B}\ . \tag{S4}$$

The critical level L_C is the threshold we use to determine whether there are any real activity present in the measurement such that:

$$\text{Cases} = \begin{cases} N_S < L_C & \text{The measurement is background.} \\ L_C < N_S & \text{There are radioactive source present.} \end{cases}$$

If the probability of making a false-positive error is 5%, L_C is set as,

$$L_C = 1.645 \cdot \sigma_{N_S} = 1.645 \cdot \sqrt{\frac{2}{A} \cdot N_B}\ . \tag{S5}$$

In the case where real radioactivity is present in the source, we introduce the detection limit N_D (higher than L_C), which is the minimum threshold of N_S such that the probability of making a false-negative error is 5%. Therefore, N_D is given by equation (S6),

$$N_D = L_C + 1.645 \cdot \sigma_{N_D} \tag{S6}$$

where σ_{N_D} is the uncertainty of N_D and follows the same equation as (S3). However, unlike the previous scenario of no real activity, $\sigma_{N_T} \neq \sigma_{N_B}$ due to the presence of radioactivity in the source. Instead, by substituting equation (S2) into (S3), σ_{N_D} can be expressed as:

$$\sigma_{N_D} = \sqrt{{\sigma_{N_T}}^2 + {\sigma_{N_B}}^2} = \sqrt{\left(\sqrt{N_T}\right)^2 + \left(\sqrt{\frac{N_B}{A}}\right)^2}$$

$$= \sqrt{\left(\sqrt{N_D + N_B}\right)^2 + \left(\sqrt{\frac{N_B}{A}}\right)^2} = \sqrt{N_D + \frac{(A+1) \cdot N_B}{A}} \,. \tag{S7}$$

By combining equations (S5), (S6), and (S7),

$$N_D = 1.645 \cdot \sqrt{\frac{2}{A} \cdot N_B} + 1.645 \cdot \sqrt{N_D + \frac{(A+1) \cdot N_B}{A}} \,. \tag{S8}$$

The minimum detectable activity MDA can be obtained by accounting for the sample's acquisition time t and detector's absolute efficiency ϵ_{abs} in the detection limit:

$$MDA = \frac{N_D}{\epsilon_{\text{abs}} \cdot t} \,. \tag{S9}$$

Text S2: Peak fitting formulation

To obtain the FWHM of the peaks, the data were fitted using CERN ROOT (version 6.19/02). The fitting function $f(x)$ consists of a summation of several gaussian functions (one gaussian function corresponding to each peak) and a constant (the baseline) which account for the background, as shown in equation (S10),

$$f(x) = \sum_{i=1}^{n} \frac{N_i}{\sigma_i \sqrt{2\pi}} \cdot e^{-\frac{1}{2}\left(\frac{x-\mu_i}{\sigma_i}\right)^2} + B \tag{S10}$$

where N_i, μ_i, and σ_i are the normalization constant, mean, and standard deviation of the ith peak. B is the background constant, which is the baseline of the graph. The fitting curve and data are presented in Figure S2.

After fitting the data according to equation S10, the fitting parameters (N_i, μ_i, σ_i, and B) were obtained. The peak resolution could be evaluated using the full-width at half maximum (FWHM), which is calculated with:

$$\mathrm{FWHM} = 2\sqrt{2\ln 2} \cdot \sigma_i\,. \tag{S11}$$

Table S2 reports the fitting parameters (μ_i and σ_i) for all the peaks identified from Figure S2. From the table, we calculated the average FWHM for the varying sample thickness. The FWHMs (in the order of increasing sample thickness) were 0.6 ± 0.3 mm, 0.9 ± 0.6 mm, and 0.4 ± 0.1 mm respectively.

Figure S1: Calibration of the thin layer sample

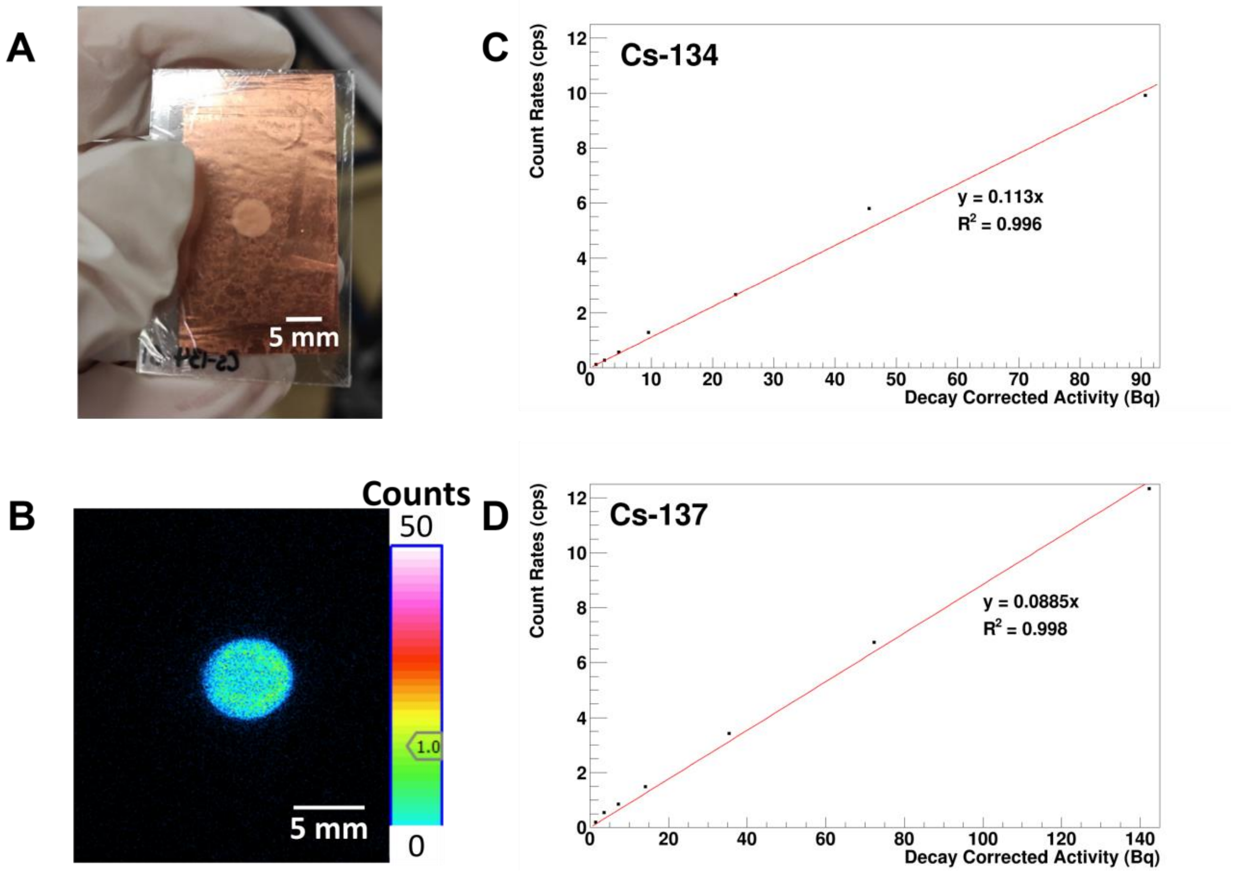

Figure S1. (A) An image of a thin layer sample (backed with copper tape), **(B)** the autoradiograph of the sample acquired with the BeaQuant system, and the activity calibration plot of **(C)** Cs-134 and **(D)** Cs-137 thin layer samples respectively. Error bars (calculated from counting statistics) are too small to be seen in the calibration plots. The fit was made to pass through the origin. Decay corrected activity was obtained from gamma spectroscopy data, which were decay corrected to the date of the BeaQuant acquisition.

Figure S2: Peak fitting for BeaQuant data with Cs-134 particle samples of varying thicknesses

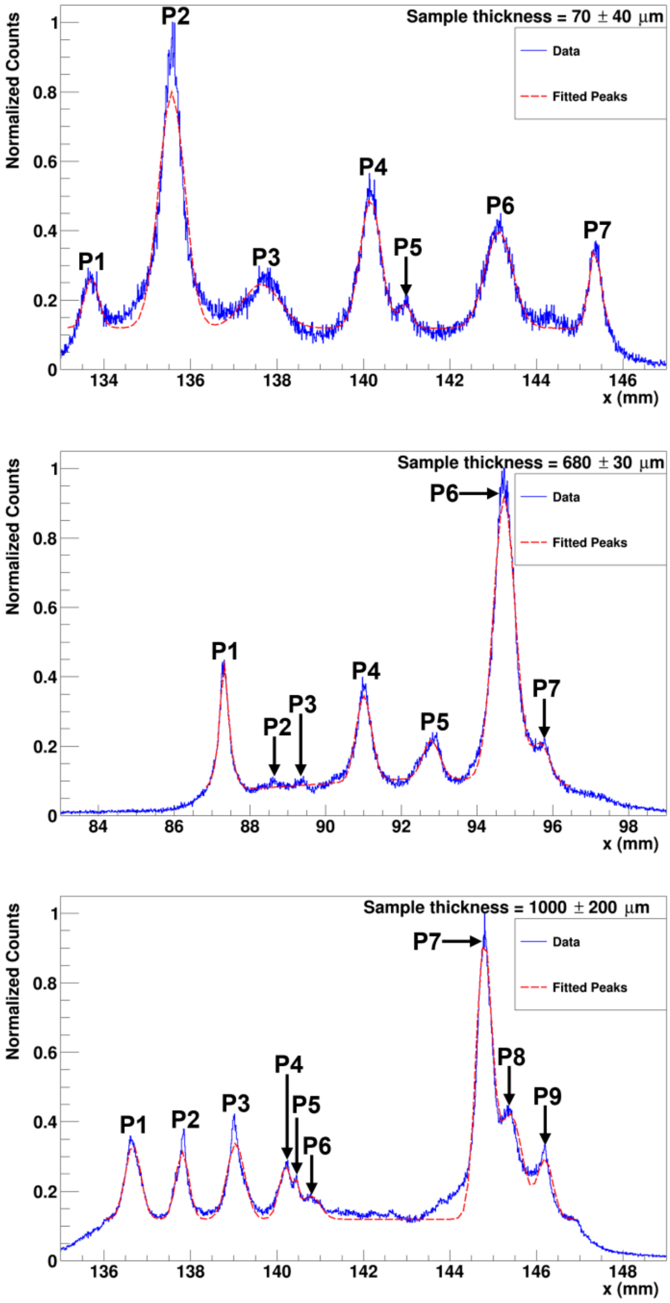

Figure S2. Peak fitting for data acquired from the BeaQuant system with Cs-134 particle samples of varying thicknesses. Counts were normalized based on the maximum peak height. The fitted peaks were identified and labelled to correspond with the fitting parameters reported in Table S2.

Figure S3: Resin embedded sample images

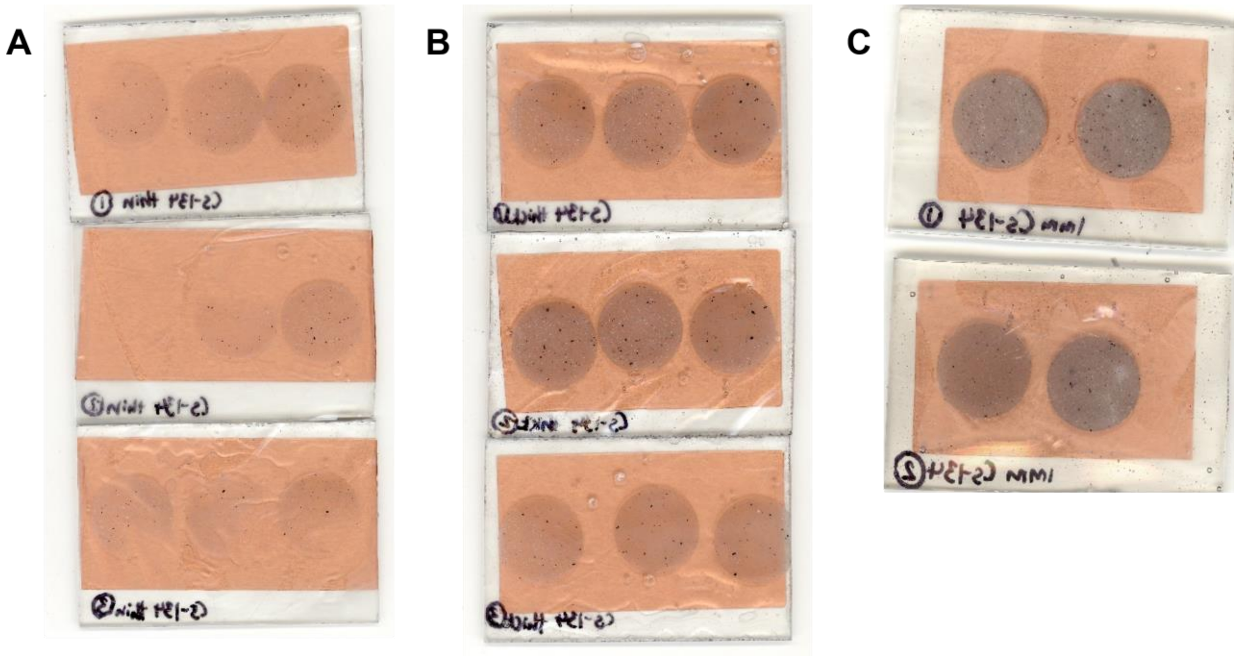

Figure S3. Images of the resin embedded samples with Cs-134 particles mixed with non-radioactive quartz. The varying sample thicknesses are **(A)** 10s of µm, **(B)** 100s of µm, and **(C)** 1 mm respectively. Copper tape was added to the back of the sample.

Figure S4: Adsorption rate plot of Cs onto Cu-HCF

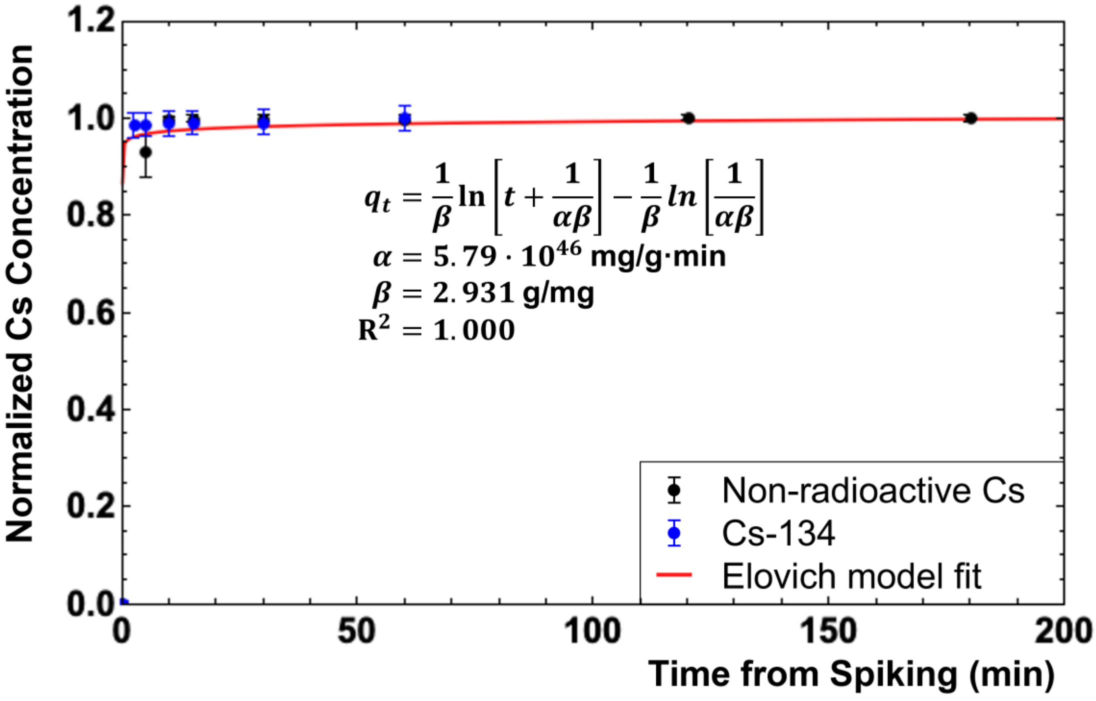

Figure S4. Adsorption rate plot of cesium (Cs) onto copper hexacyanoferrate at pH 7, room temperature, and pressure. The adsorption of non-radioactive Cs was carried out for a total time of 1440 minutes, as an initial pilot test for radiation safety. The data for the non-radioactive Cs (in black) were obtained by measuring aliquots of different time points with the inductively coupled plasma-mass spectrometry. The error bars represent the calculated standard error from triplicates. Results from the non-radioactive Cs sorption were fitted to the Elovich model (red line). The Cs-134 adsorption was carried out for a total time of 60 minutes. Cs-134 sorption data (in blue) were obtained by measuring the aliquots of different time points with gamma spectroscopy. The error bars were calculated from counting statistics. This Cs-134 data was not fitted to the Elovich model due to insufficient data points. Both plots were normalized against the maximum concentration for easier comparison.

Figure S5: Geometrical visualization of the Monte Carlo simulation with GEANT4

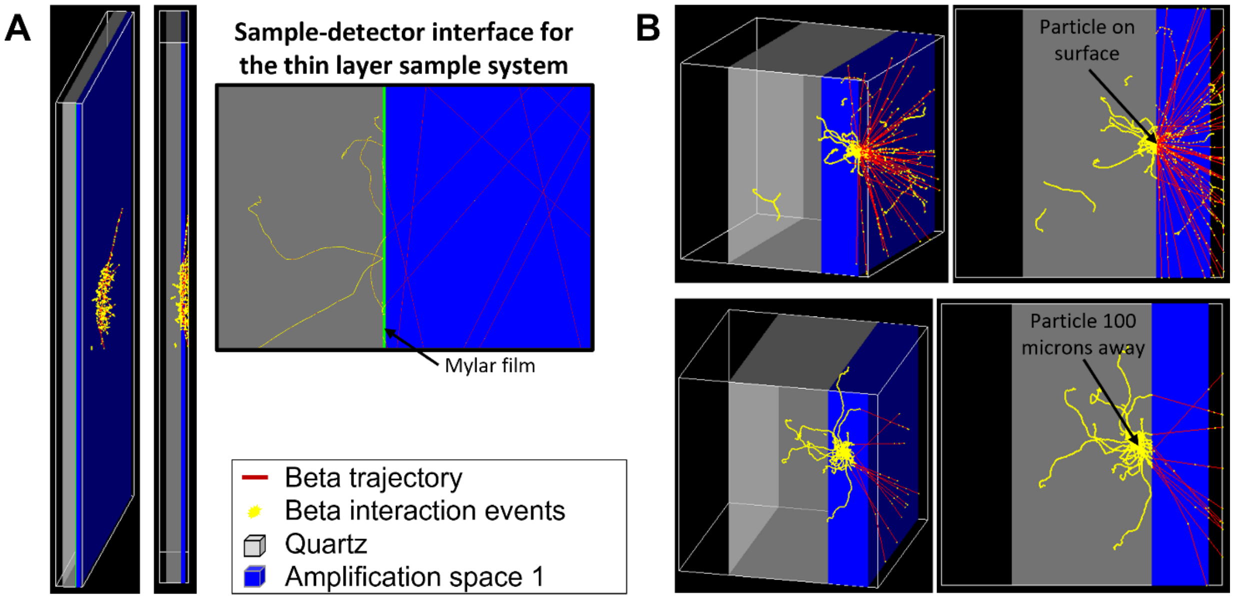

Figure S5. Geometrical visualization of the Monte Carlo simulation with GEANT4. GEANT4 geometry of the **(A)** thin layer sample (25 mm^2 surface area, planar) with a 3 µm thick mylar on the sample surface, and **(B)** particulate Cs-134 (10 µm radius, spherical) in homogeneous quartz (2.65 g/cm^3) in varying depths. In each image, 100 decay events were shown. Dimensions of the quartz for (A) was set to 30 × 45 × 1.2 mm, which matches the glass slides used in the experiment. Dimensions of the quartz for (B) was 1 × 1 × 1 mm. The gas defined in amplification space 1 was Ne(90%)CO_2(10%) in a pressure of 1.1×10^5 Pa.

Table S1: Energies and intensities of electrons emitted by Cs-134 and Cs-137

Table S1. Energies and intensities of the electrons emitted by Cs-134 and Cs-137. This table only report electrons with intensity of > 0.1%.

Radioisotope	Emission type	Energy (keV)	Intensity (%)
Cs-134	β^-	88.8 ± 0.4*	27.27 ± 0.03
		415.4 ± 0.4*	2.499 ± 0.009
		658.1 ± 0.4*	70.17 ± 0.07
	Auger	3.67	0.851 ± 0.006
	Conversion electron	531.890 ± 0.003	0.12544 ± 0.00014
		567.2804 ± 0.0020	0.4910 ± 0.0006
		758.423 ± 0.004	0.22049 ± 0.00015
Cs-137	β^-	513.97 ± 0.17*	94.70 ± 0.20
		1175.63 ± 0.17*	5.30 ± 0.20
	Auger	3.67	7.40 ± 0.10
		26.4	0.78 ± 0.03
	Conversion electron	624.216 ± 0.003	7.79 ± 0.11
		655.668 ± 0.003	1.402 ± 0.020
		660.364 ± 0.003	0.300 ± 0.004

*Energy for beta emission refers to the end-point energy.

Table S2: Summary of fitting parameters and FWHM for each peak

Table S2. Summary of fitting parameters and FWHM for each peak. The error value for μ is derived from the pixel size of the autoradiograph (0.01 mm), whereas the error values for σ and FWHM come from the fitting error obtained with CERN ROOT.

Sample thickness (µm)	Peak	µ (mm)	σ (mm)	FWHM (mm)
	P1	133.71 ± 0.01	0.162 ± 0.004	0.382 ± 0.010
	P2	135.57 ± 0.01	0.300 ± 0.003	0.706 ± 0.007
	P3	137.64 ± 0.01	0.430 ± 0.012	1.010 ± 0.030
70 ± 40	P4	140.16 ± 0.01	0.241 ± 0.003	0.568 ± 0.007
	P5	140.96 ± 0.01	0.137 ± 0.009	0.323 ± 0.022
	P6	143.13 ± 0.01	0.315 ± 0.004	0.742 ± 0.010
	P7	145.35 ± 0.01	0.135 ± 0.003	0.318 ± 0.007
	P1	87.32 ± 0.01	0.083 ± 0.003	0.195 ± 0.008
	P2	88.65 ± 0.01	0.840 ± 0.060	1.980 ± 0.150
	P3	89.31 ± 0.01	0.470 ± 0.030	1.107 ± 0.071
680 ± 30	P4	91.01 ± 0.01	0.205 ± 0.002	0.482 ± 0.005
	P5	92.76 ± 0.01	0.535 ± 0.005	1.260 ± 0.012
	P6	94.72 ± 0.01	0.290 ± 0.001	0.684 ± 0.003
	P7	95.68 ± 0.01	0.244 ± 0.005	0.574 ± 0.011
	P1	136.67 ± 0.01	0.178 ± 0.001	0.420 ± 0.003
	P2	137.81 ± 0.01	0.149 ± 0.001	0.350 ± 0.003
	P3	139.05 ± 0.01	0.201 ± 0.002	0.474 ± 0.004
	P4	140.20 ± 0.01	0.150 ± 0.001	0.353 ± 0.001
1000 ± 200	P5	140.47 ± 0.01	0.059 ± 0.002	0.140 ± 0.006
	P6	140.80 ± 0.01	0.200 ± 0.002	0.471 ± 0.001
	P7	144.79 ± 0.01	0.182 ± 0.001	0.430 ± 0.002
	P8	145.41 ± 0.01	0.264 ± 0.002	0.622 ± 0.005
	P9	146.20 ± 0.01	0.179 ± 0.002	0.422 ± 0.004

Table S3: Measured thickness of the resin embedded samples

Table S3. The measured thickness of the resin embedded samples. The error values come from the standard deviation of 10 thickness readings per sample slide.

Sample		Measured Sample Thickness (µm)
**Approximate thickness**	**Replicates**	
10s of µm	1	70 ± 40
	2	40 ± 20
	3	50 ± 30
100s of µm	1	680 ± 60
	2	680 ± 30
	3	580 ± 110
1 mm	1	1000 ± 200
	2	1000 ± 200
